# Small-molecule inhibitor of AF9/ENL-DOT1L/AF4/AFF4 interactions suppresses malignant gene expression and tumor growth

**DOI:** 10.7150/thno.56737

**Published:** 2021-07-13

**Authors:** Fangrui Wu, Shenyou Nie, Yuan Yao, Tong Huo, Xin Li, Xiaowei Wu, Jidong Zhao, Yi-Lun Lin, Yinjie Zhang, Qianxing Mo, Yongcheng Song

**Affiliations:** 1 Department of Pharmacology and Chemical Biology, Baylor College of Medicine, 1 Baylor Plaza, Houston, TX 77030, USA.; 2 Dan L. Duncan Comprehensive Cancer Center, Baylor College of Medicine, 1 Baylor Plaza, Houston, TX 77030, USA.; 3 H. Lee Moffitt Cancer Center & Research Institute, 12902 USF Magnolia Drive, Tampa, FL 33612, USA.

**Keywords:** MLL-rearranged leukemia, Super elongation complexes, Protein-protein interaction, Small-molecule inhibitor, Cancer therapeutics

## Abstract

Chromosome translocations involving mixed lineage leukemia (MLL) gene cause acute leukemia with a poor prognosis. MLL is frequently fused with transcription cofactors AF4 (~35%), AF9 (25%) or its paralog ENL (10%). The AHD domain of AF9/ENL binds to AF4, its paralog AFF4, or histone-H3 lysine-79 (H3K79) methyltransferase DOT1L. Formation of AF9/ENL/AF4/AFF4-containing super elongation complexes (SEC) and the catalytic activity of DOT1L are essential for MLL-rearranged leukemia. Protein-protein interactions (PPI) between AF9/ENL and DOT1L/AF4/AFF4 are therefore a potential drug target.

**Methods**: Compound screening followed by medicinal chemistry was used to find inhibitors of such PPIs, which were examined for their biological activities against MLL-rearranged leukemia and other cancer cells.

**Results**: Compound-**1** was identified to be a novel small-molecule inhibitor of the AF9/ENL-DOT1L/AF4/AFF4 interaction with IC50s of 0.9-3.5 µM. Pharmacological inhibition of the PPIs significantly reduced SEC and DOT1L-mediated H3K79 methylation in the leukemia cells. Gene profiling shows compound-**1** significantly suppressed the gene signatures related to onco-MLL, DOT1L, HoxA9 and Myc. It selectively inhibited proliferation of onco-MLL- or Myc-driven cancer cells and induced cell differentiation and apoptosis. Compound-**1** exhibited strong antitumor activity in a mouse model of MLL-rearranged leukemia.

**Conclusions:** The AF9/ENL-DOT1L/AF4/AFF4 interactions are validated to be an anticancer target and compound-**1** is a useful in vivo probe for biological studies as well as a pharmacological lead for further drug development.

## Introduction

Chromosome translocations involving mixed lineage leukemia (MLL, also known as MLL1 or KMT2A) gene cause ~75% of infant and ~10% child/adult acute leukemias, characterized to be either acute myeloid leukemia (AML) or acute lymphocytic leukemia (ALL) 1-3. Compared to most other pediatric ALL with a 5-year survival of ~90%, MLL-rearranged (MLL-r) ALL has a particularly poor prognosis with 5-year survival of <40% 4-6. For very young infants, the survival is even < 20% 7. Similar to other AMLs, MLL-r AML patients also have low 5-year survival rates of ~45% 8.

The N-terminal ~1400 residues of MLL contain DNA-interacting AT-hooks and CxxC domains, which is merged with one of >70 proteins to form a fusion oncogene. However, the most frequent partners are transcription cofactors AF9 (~25%) and its paralog ENL (10%), AF4 (35%) and ELL (5%) 1, 2, 9. Together with cyclin-T1 dependent kinase P-TEFb, these proteins associate with each other and are essential members of several isolated transcription elongation complexes, such as super elongation complex (SEC) 10-12.

**Figure 1:**
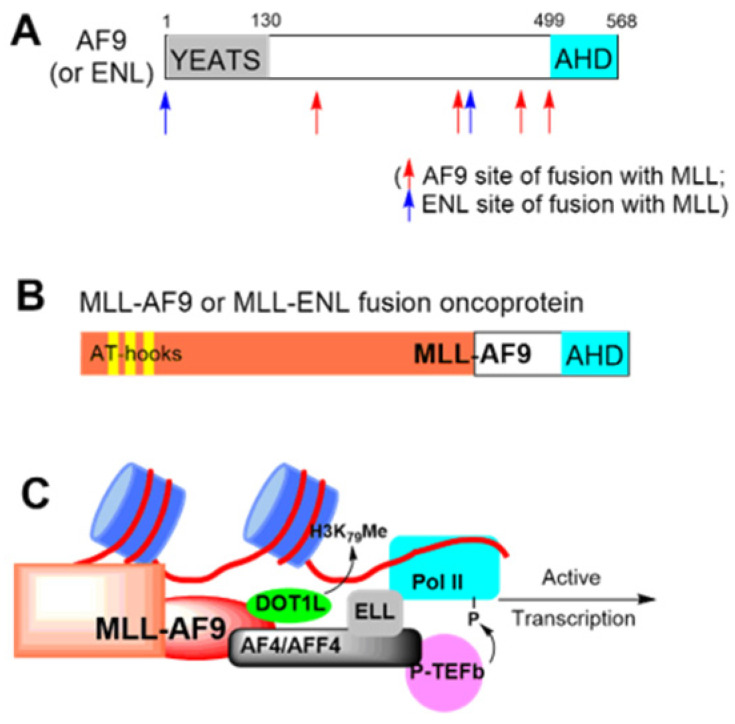
Illustration of (**A**) AF9 or ENL protein, (**B**) MLL-AF9/-ENL fusion oncoprotein, and (**C**) Functions of MLL-AF9. MLL binds to its target gene promoters, with the AHD domain of AF9 recruiting DOT1L for H3K79 methylation, or AF4/AFF4, ELL and P-TEFb for transcription elongation.

AF9 and ENL contain a YEATS and AHD domain (Figure 1A). While YEATS is lost in most clinical variances of MLL-AF9/-ENL, the AHD domain is required and plays a pivotal role in MLL-r leukemia (Figure 1B) 11, 13. In MLL-AF9/-ENL leukemia, MLL functions as a transcription factor to recognize its target genes, while AHD binds to AF4 or its paralog AFF4 (Figure 1C) 11, 14. AF4/AFF4 form a heterodimer 15, 16 and further recruit P-TEFb and ELL 17, 18. P-TEFb phosphorylates RNA polymerase II (Pol II), and ELL binds to Pol II and enhances its activity 19, 20. These two events are key steps for Pol II to leave promoter-proximal pausing and start transcription elongation. Protein-protein interactions (PPI) between AF9/ENL's AHD and AF4/AFF4 are essential for the formation of SEC and required for onco-MLL-mediated leukemogenesis 11, 12. Moreover, SEC also regulates expression of transcription factor Myc, a master regulator for cancer cell growth, and HIV, implicating such PPIs' roles in other cancers and HIV infection 21.

AF9/ENL AHD also binds to histone-H3 lysine-79 (H3K79) methyltransferase DOT1L 22, 23 (Figure 1C). DOT1L is a therapeutic target for MLL-r leukemia: genome-wide hypermethylation of H3K79 is characteristic to the cancer 24 and DOT1L's catalytic activity is required for expression of MLL-target genes, such as HoxA9 and Meis1. Pharmacological inhibition of DOT1L has been vigorously developed and an advanced inhibitor is in clinical trials against the leukemia 25, 26.

NMR studies show while AF9 AHD alone is disordered, it binds to a consensus peptide of LxVxIxLxxV/L in DOT1L or AF4/AFF4 to form a structured protein complex (Supplementary Materials Figure S1) 13, 14. In addition, high affinity binding of AF9 AHD to DOT1L is required for oncogenesis 13. Therefore, PPIs between AHD and DOT1L or AF4/AFF4 are a potential drug target for MLL-r leukemia. There are no small-molecule inhibitors of such PPIs. A series of 7-mer DOT1L peptidomimetic compounds were reported to disrupt the PPIs biochemically, but no cellular activities were disclosed 27. Here, we report the discovery and biological activities of a small-molecule compound **1** that disrupts the PPIs between the AHD domain of AF9/ENL and DOT1L or AF4/AFF4. It significantly inhibits malignant gene expression and proliferation of MLL-r leukemia and Myc-driven cancer.

## Results

### Discovery of inhibitors of the AHD domain of AF9

**Figure 2:**
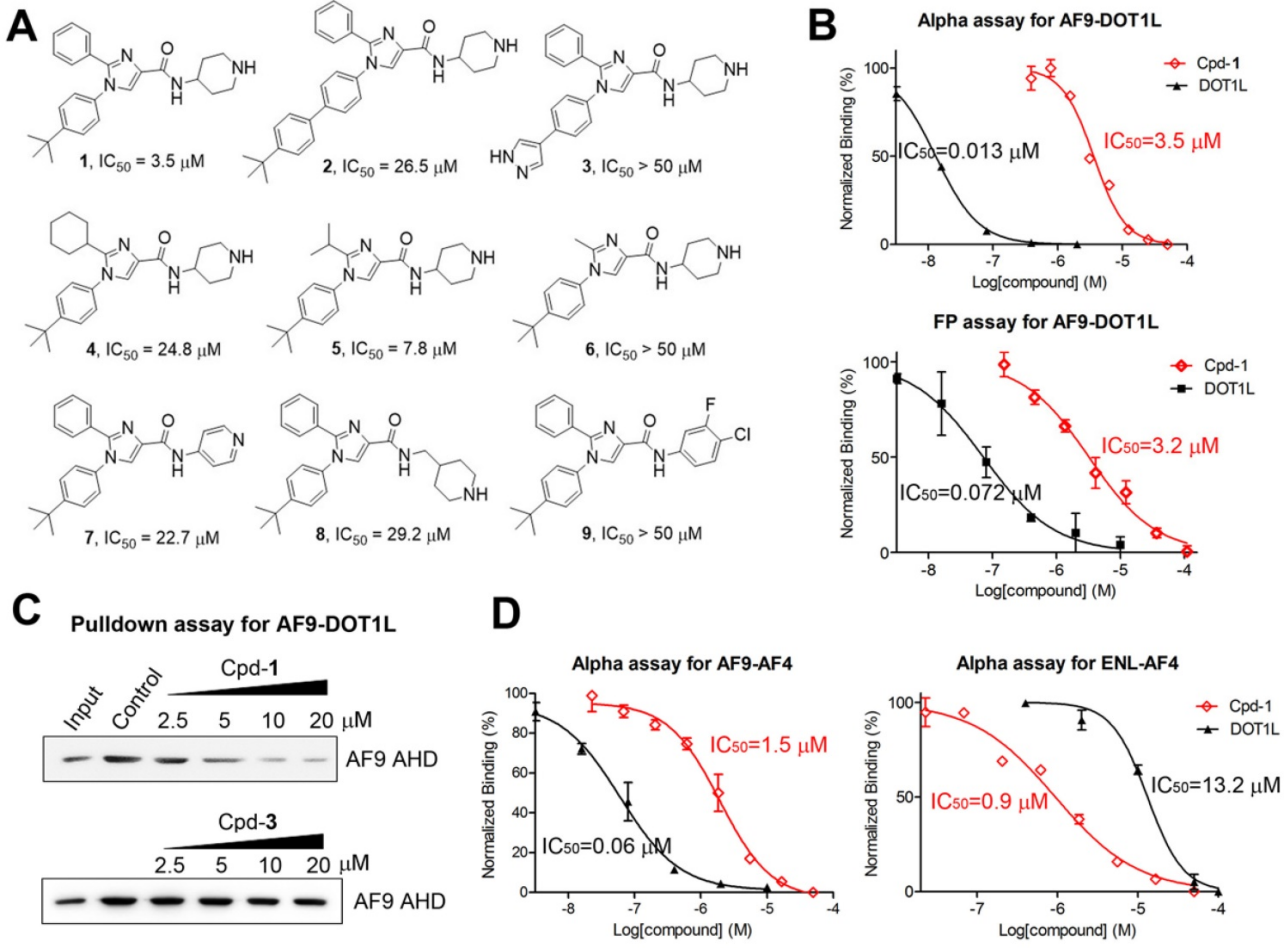
Biochemical characterization of inhibitors of the AHD domain of AF9/ENL. (**A**) Inhibitor structures and activities; (**B**) Dose-response curves of Cpd-**1** and DOT1L-peptide inhibiting the AF9-DOT1L interaction; (**C**) Pulldown assays showing Cpd-**1** dose-dependently reduced the amounts of AF9 AHD bound to DOT1L coated resins, while inactive Cpd-**3** cannot. (Input: purified AF9 AHD; Control: untreated samples); (**D**) Dose-response curves of Cpd-**1** and DOT1L inhibiting the AF9/ENL-AF4 interaction.

We developed an Alpha (amplified luminescent proximity homogeneous assay) assay to determine the binding and inhibition of the PPI between maltose-binding protein (MBP)-tagged AF9 AHD and a biotin-labeled DOT1L peptide, using AlphaLisa streptavidin donor beads and anti-MBP acceptor beads. Laser excitation to the donor beads generates singlet oxygen radicals, which can travel for <200 nm in solution and cannot reach the free acceptor beads. The PPI brings the DOT1L coated donor beads and AHD coated acceptor beads together, allowing the radicals to activate the acceptor beads and produce luminescence. Its intensity is reduced dose-dependently when an inhibitor, such as non-biotinylated DOT1L (IC_50_ = 13 nM, Figure 2B and Table 1), disrupts the PPI. We also developed a fluorescence polarization (FP) assay. A fluorescein-labeled DOT1L peptide has a higher freedom in solution with a lower polarization value. With increasing concentrations of AF9 AHD that binds to DOT1L, the polarization increased with a *K*_d_ (dissociation constant) of 87 nM (Figure S2A), while unlabeled DOT1L can reduce it (Figure 2B, IC_50_ = 72 nM).

We screened our proprietary library of ~1,500 compounds synthesized for targeting several proteins with a peptide substrate, including H3K4 demethylase LSD1 28, histone acetyltransferase p300 29 and flavivirus proteases 30, with a rationale that there is a higher likelihood to find AHD inhibitors from these peptide-competitive compounds. Imidazole-4-carboxamide compound **2** (Figure 2A) was found to be an inhibitor with IC_50_ of ~25 µM in both assays. Medicinal chemistry studies yielded compound **1** which inhibited the AF9 AHD-DOT1L interactions with an IC_50_ of 3.5 µM or 3.2 µM using Alpha or FP assay (Figure 2A/B and Table 1). Structures and activities of representative compounds are summarized in Figure 2A, showing structure-activity relationships. For R1, the hydrophobic 4-*tert*-butylphenyl group in compound **1** is more favorable than the larger group in **2** or the more polar pyrazole in **3** (IC_50_ > 50 µM). With the activity order of -Me < -*i*-Pr < -Ph in compounds **6**, **5** and **1**, a small R2 substituent is disfavored. However, the cyclohexyl R2 substituent in compound **4** seems to be too bulky, showing a reduced activity (IC_50_ = 24.8 µM) as compared to **1** with a phenyl. For R4, the basic piperidine in **1** that is protonated at physiological pH is more active than the less basic pyridine in compound **7** (IC_50_ = 22.7 µM). Compound **8** (IC_50_ = 29.2 µM) with a longer R4 is considerably less active, while compound **9** without a terminal N atom is inactive.

A non-optical pull-down assay was used to further validate compound **1**. DOT1L-coated beads were incubated with AF9 AHD in the presence of increasing concentrations of **1**. Upon washing, Western blot showed that compound **1** reduced beads-bound AF9 AHD with a similar IC_50_, while inactive compound **3** cannot (Figure 2C).

Overall, three independent biochemical assays show compound **1** is an inhibitor of the AF9-DOT1L interaction. Moreover, compound **1** did not significantly inhibit DOT1L, LSD1 and p300 at 100 µM (Figure S3), showing a high selectivity.

### Compound 1 inhibits PPIs between AF9/ENL and AF4/AFF4

ENL is also a major MLL fusion partner (~10%). The AHD domains of human ENL and AF9 share a high homology with 82% identity and 89% similarity (Figure S1A). Similar Alpha and FP assays were developed for the PPIs between AF9/ENL and AF4 or AFF4 peptide, as summarized in Table 1. Non-biotinylated DOT1L was found to inhibit the AF9-AF4 and -AFF4 interactions with an IC_50_ of 60 and 95 nM, respectively (Figure 2D). It exhibited significantly lower activities against the ENL-AF4/-AFF4 interactions with IC_50_ of 13.2 and 2.0 µM. Compound **1** inhibited AF9-AF4 and -AFF4 with IC_50_ of 1.5 and 2.5 µM, respectively. It exhibited comparable activity against the ENL and AF4 or AFF4 interactions with IC_50_ of 0.94 (Figure 2D) and 3.3 µM. Our attempts to develop an Alpha assay for ENL-DOT1L failed. Moreover, due to their weaker binding affinity (*K*_d_: 640 nM, Table 1), the FP assay using 1 µM of ENL (as compared to 0.1 µM of AF9 used in the AF9 FP assay) is unsuitable for determining the IC_50_ of a strong inhibitor. Compound **1**'s activity against ENL-DOT1L was therefore not determined.

Our results show that compound **1** is a novel, broadly active small-molecule inhibitor of the PPIs between AF9/ENL and AF4, AFF4 or DOT1L with an IC_50_ of 0.94-3.5 µM.

### Compound 1 inhibits cellular AF9/ENL-DOT1L/AF4/AFF4 interactions

Cell-based assays were used to characterize biological activities of compound **1** in MLL-r leukemia cell lines Molm-13 and MV4-11 harboring MLL-AF9 and MLL-AF4 fusions, respectively. Compound **3**, which is inactive for these PPIs, was included as a negative control.

**Figure 3:**
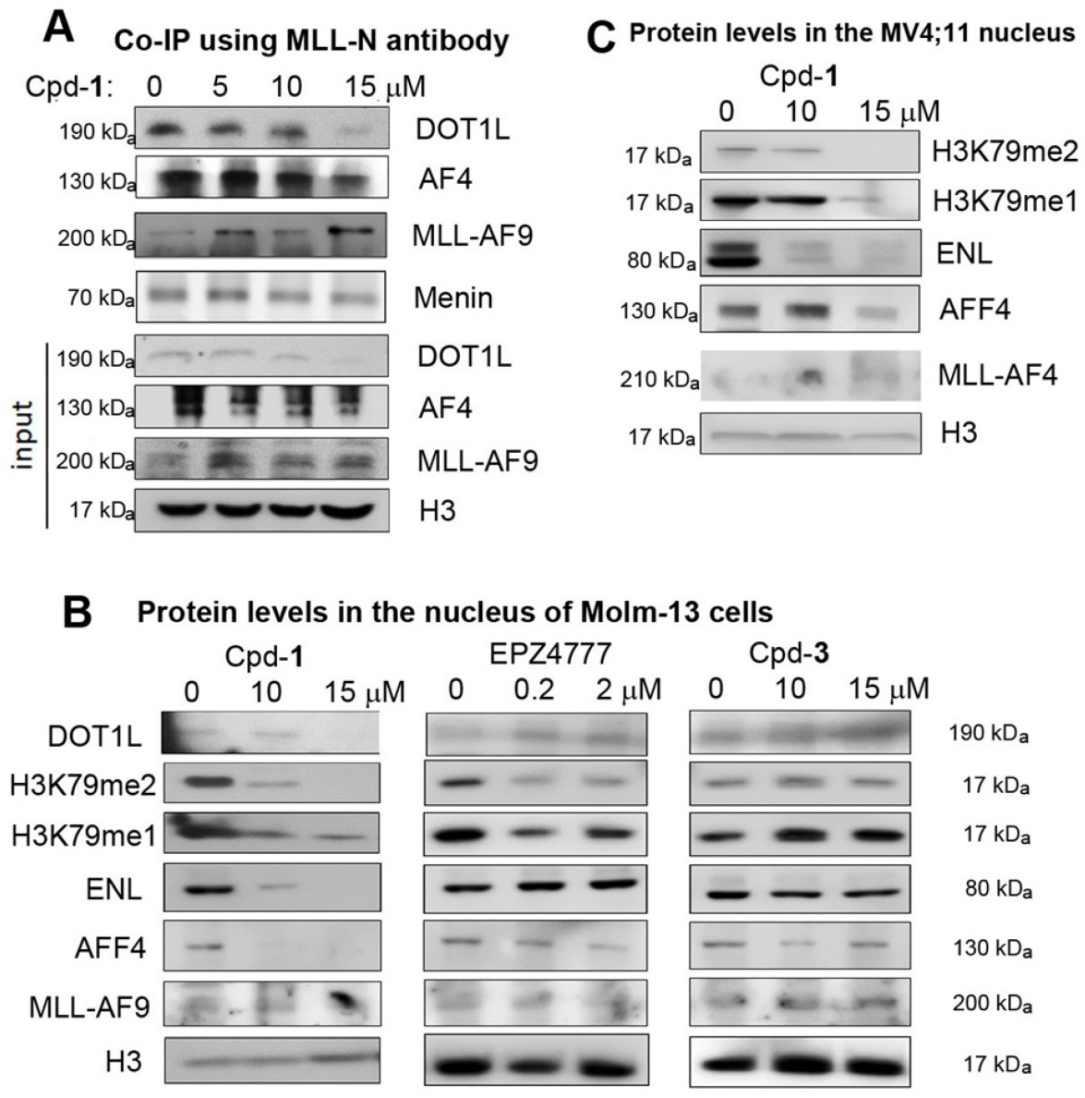
Cellular assays showing Cpd-**1** inhibited the PPIs between AF9/ENL and DOT1L, AF4 or AFF4. (**A**) Co-immunoprecipitation using an antibody against the N-terminal MLL shows treatment of Molm-13 cells with Cpd-**1** for 2 days dose-dependently disrupted the PPIs between MLL-AF9 and DOT1L or AF4; Upon incubation for 3 days, (**B**) Cpd-**1** reduced the nuclear levels of DOT1L, H3K79me1 and me2, ENL and AFF4 in Molm-13 cells, while inactive Cpd-**3** did not. A DOT1L inhibitor EPZ4777 only reduced those of H3K79me1 and me2, but not other proteins; (**C**) Cpd-**1** reduced the nuclear protein levels of H3K79me1 and me2 and ENL in MV4;11 cells.

Co-immunoprecipitation using an antibody against N-terminal MLL shows treatment of Molm-13 cells with compound **1** inhibited the PPIs between MLL-AF9 and DOT1L or AF4, resulting in dose-dependent decrease of MLL-AF9-bound DOT1L and AF4 (Figure 3A). These results indicate compound **1** is cell permeable and can disrupt the AF9-DOT1L or -AF4 interaction in cells. Next, we investigated how compound **1** affects these transcription cofactors in the nucleus, where they regulate gene expression. As shown in Figure 3B, while the nuclear levels of MLL-AF9 seem unaffected, treatment of Molm-13 cells with **1** significantly reduced DOT1L and its products H3K79-Me2 and H3K79-Me1 in a dose-dependent manner. ENL and AFF4 were also significantly decreased. Inactive compound **3** did not change these protein levels in the nucleus. DOT1L inhibitor EPZ4777 25 reduced the methylation levels of H3K79, but not other proteins (Figure 3B). In addition, the cytoplasmic levels of these proteins were not significantly affected by the treatment of compound **1** (Figure S4), except for ENL at 15 μM of Cpd-**1**. These results support due to its ability to disrupt the AF9-DOT1L interaction, compound **1** can decrease MLL-AF9-mediated recruitment of DOT1L into the chromatin, which caused reduced H3K79 methylation. In this regard, despite lack of activity against DOT1L, compound **1** can mimic EPZ4777 to reduce H3K79 methylation. Additionally, because of its ability to disrupt the AF9/ENL-AF4/AFF4 interactions, compound **1** inhibited MLL-AF9 mediated recruitment of SEC, as evidenced by significantly reduced ENL and AFF4, two essential members of SEC.

Similarly, compound **1** dose-dependently reduced the nuclear levels of H3K79-Me1/2 and ENL in MLL-AF4 driven MV4;11 leukemia cells (Figure 3C), while it did not cause significantly decreased AFF4 at 10 μM, presumably due to direct recruitment of AFF4 by MLL-AF4 through heterodimerization. Nonetheless, it is clear that disrupting AF9/ENL AHD's PPIs by compound **1** can also inhibit MLL-AF4 mediated recruitment of DOT1L and ENL.

### Compound 1 suppresses expression of characteristic genes in MLL-r leukemia

**Figure 4:**
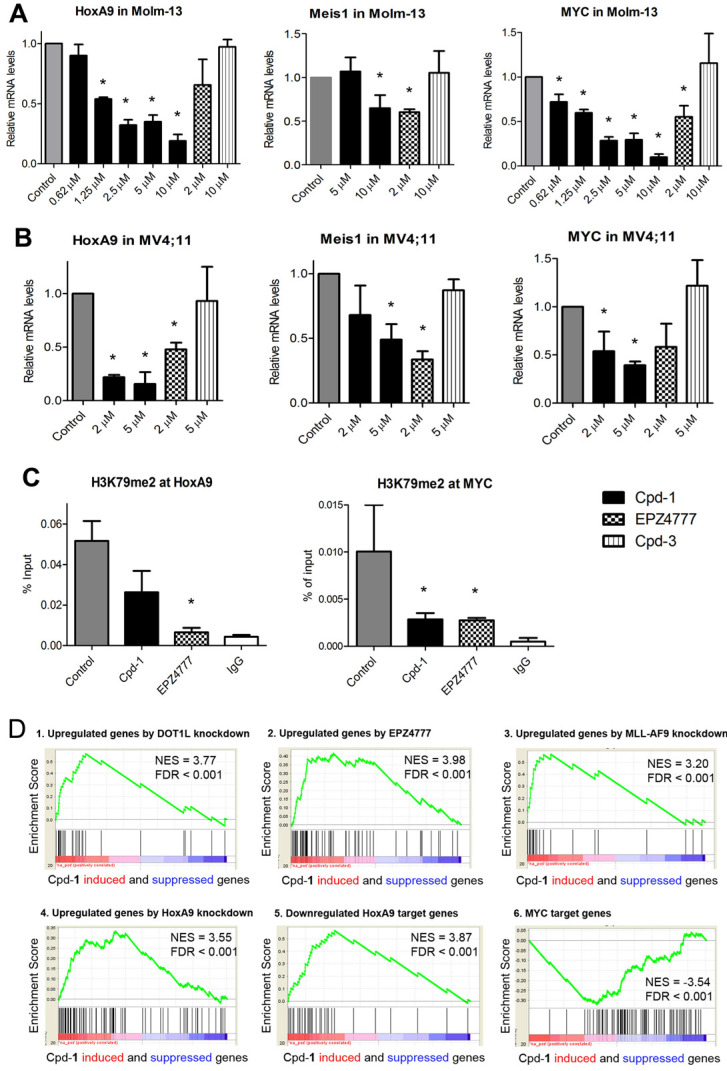
Cpd-**1** inhibited aberrant gene expression in MLL-r leukemia. (**A**,** B**) Treatment of (**A**) Molm-13 and (**B**) MV4;11 cells with Cpd-**1** for 4 days dose-dependently suppressed expression of MLL target genes HoxA9, Meis1 and Myc (* *p* < 0.05). DOT1L inhibitor EPZ4777 behaved similarly, but inactive Cpd-**3** had no activity. Data were from two or more experiments; (**C**) Similar to EPZ4777 (2 μM), treatment with Cpd-**1** (5 μM for 4 days) caused decreased levels of H3K79me2 in the gene promoters of HoxA9 and Myc in Molm-13 cells (* *p* < 0.05); (**D**) Gene profiling followed by gene set enrichment analysis (GSEA) shows that treatment of Molm-13 cells with Cpd-**1** (5 µM for 4 days) recapitulated activities of 1) DOT1L knockdown (GSE25911), 2) DOT1L inhibition by EPZ4777 (GSE29828), 3) MLL-AF9 knockdown (GSE36592), and 4) HoxA9 knockdown (GSE33518). It also significantly 5) upregulated HoxA9-downregulated target genes (GSE13714), and 6) downregulated Myc target genes (GSE32220).

Next, compound **1** was tested for its ability to change expression of characteristic MLL-r leukemia genes HoxA9, Meis1 and Myc. Myc is also overexpressed in a broad range of AML and other cancers 31. Similar to EPZ4777, treatment with compound **1** significantly inhibited expression of HoxA9, Meis1 and Myc (Figure 4A/B), while inactive compound **3** had no activity. In addition, using chromatin immunoprecipitation (ChIP) followed by qPCR, treatment with **1** significantly reduced H3K79-Me2 in the promoters of HoxA9 and Myc, mimicking the activity of EPZ4777 (Figure 4C). However, it did not significantly reduce H3K79-Me2 in a non-transcribed DNA region 32 (Figure S5), presumably because SEC is not recruited there. These results are consistent with compound **1**'s activity to reduce DOT1L/H3K79 methylation, ENL and AFF4 in MLL target genes, all of which are required for malignant gene expression in MLL-r leukemia.

### Compound 1 suppresses the gene signatures of MLL-r leukemia

RNA-sequencing was performed to investigate how **1**-mediated disruption of the PPIs between AF9/ENL and DOT1L or AF4/AFF4 affects global gene expression in MLL-r leukemia. RNAs from the control and compound **1** (5 µM) treated Molm-13 cells were extracted and sequenced. Gene set enrichment analysis (GSEA) showed that compound **1** caused significant upregulation of a gene set that was upregulated upon DOT1L knockdown 33, with normalized enrichment score (NES) of 3.77 and false discovery rate (FDR) of < 0.001 (Figure 4D.1), indicating treatment with **1** caused similar gene expression changes to DOT1L knockdown. Treatment with **1** recapitulated the expression pattern of DOT1L inhibition by EPZ4777 25 (Figure 4D.2, NES = 3.98, FDR < 0.001). Compound **1** significantly upregulated gene sets that were upregulated upon knockdown of MLL-AF9 and HoxA9 34, indicating the compound treatment mimics knockdown of these two onco-proteins (Figure 4D.3 and 4). In addition, compound **1** suppressed expression of HoxA9- and Myc-target gene sets: it upregulated HoxA9-downregulated genes 35 (NES = 3.87, FDR < 0.001) and downregulated Myc-target genes 36 (NES = -3.54, FDR < 0.001) (Figure 4D.5 and 6). Overall, gene profiling results show compound **1** significantly suppressed the gene signatures related to DOT1L, MLL-AF9, HoxA9 and Myc in Molm-13 cells.

### Cpd-1 inhibited cell proliferation, induced differentiation and apoptosis of MLL-r leukemia

**Figure 5:**
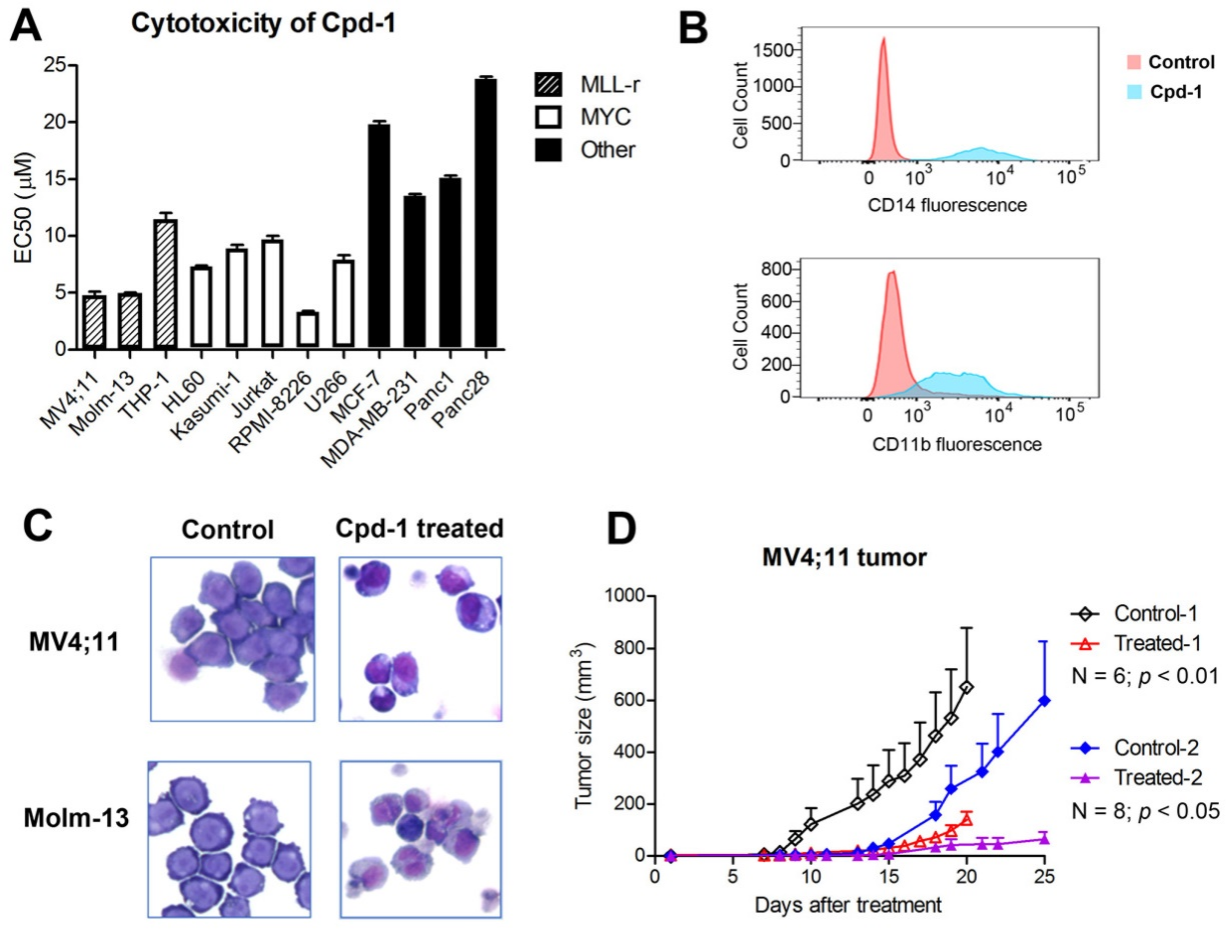
Cpd-**1** inhibited proliferation and induced differentiation of MLL-r leukemia. (**A**) Upon incubation for 7 days, Cpd-**1** inhibited proliferation of MLL-r leukemia (MC4;11, Molm-13 and THP-1) and Myc-driven blood cancer (the middle panel) cells with EC_50_ of 3-11 µM, while it had less activity against other tumor cells (the right panel); (**B**) Treatment of Molm-13 cells with Cpd-**1** (5 μM for 5 days) led to significantly more cells expressing high levels of CD14 and CD11b, cell surface proteins for macrophages/monocytes; (**C**) Giemsa staining shows upon treatment with Cpd-**1** (5 μM for 5 days), leukemia cells exhibited a more differentiated phenotype with reduced nucleus; (**D**) Treatment with Cpd-**1** (15 mg/kg for 10 days) significantly inhibited tumor growth in two cohorts of mice with subcutaneously xenografted MV4;11 leukemia.

Compound **1** exhibited strong antitumor activities with EC_50_s of 4.7-11 µM against proliferation of MLL-r leukemia cells Molm-13, MV4;11 and THP-1 (with MLL-AF9) (Figure 5A, Figure S6 and Table S1). Myc-driven blood cancer cells, including AML cells HL60 and Kasumi-1, ALL cells Jurkat, and multiple myeloma cells RPMI8226 and U266, were also susceptible to **1** with EC_50_s of 3.3-9.7 µM. Compound **1** showed reduced activity against MCF-7 (ER+ breast), MDA-MB-231 (triple-negative breast) and two pancreatic cancer cells. Inactive compound **3** did not inhibit proliferation of these cancer cells (EC_50_ > 50 µM). The differential antiproliferation activities of compound **1** is consistent with its ability to suppress DOT1L/H3K79 methylation and SEC regulated gene expression, which are critical to MLL-r leukemia and Myc-driven blood cancer, but largely dispensable to other solid tumor cells. It is noted that, similar to many epigenetic inhibitors (e.g., DOT1L or LSD1 inhibitors 25, 37, 38), compound **1** did not significantly inhibit proliferation of sensitive cancer cells during the first 2-3 days, while it showed potent activity upon incubation for more than 5 days (Figure S7). This slow action seems to be common for compounds, such as compound **1** and epigenetic inhibitors, that do not have general cytotoxicity (e.g., inhibiting DNA/protein synthesis), but inhibit aberrant gene expression in cancer (Figure 4).

Treatment of Molm-13 cells for 5 days with compound **1** at 10 and 15 µM caused significant apoptosis of 44.2% and 73.6%, respectively (Figure S8), while a short-term, 2- or 3-day incubation did not cause significant apoptosis (< 5%). Compound **1** (5 μM for 5 days) induced significant differentiation, with more cell populations expressing CD14 and CD11b, two cell surface proteins for macrophages/monocytes (Figure 5B). Upon treatment, MLL-r leukemia cells also exhibited a more differentiated phenotype with reduced nucleuses (Figure 5C).

### Cpd-1 exhibited strong antitumor activity in a mouse model of MLL-r leukemia

In vivo antitumor activity of compound **1** was evaluated in a commonly used mouse model of MLL-r leukemia 25, 26. 10^7^ MV4;11 cells in 0.1 mL of saline and Matrigel (1:1) were injected subcutaneously into NOD-SCID mice, which can develop palpable tumors in ~2 weeks. As shown in Figure 5D and Figure S9, treatment with compound **1** (15 mg/kg for Day 1-10) significantly inhibited tumor growth in two cohorts of mice (N = 6 and 8; *p* < 0.01 and 0.05, respectively), while such dose of compound **1** did not cause significant weight losses (Figure S10) or other overt toxicity to the animals.

## Discussion

MLL-r leukemia accounts for ~75% of acute leukemia in infants and ~10% in children and adults with a poor prognosis. Current treatments are conventional chemotherapeutics, which non-selectively kill rapidly proliferating cells including normal stem/progenitor cells. This causes toxicities and side effects and limits clinical efficacy. More effective therapies are needed, such as targeted therapeutics inhibiting a protein critical to MLL-r leukemia but dispensable to normal cells.

Despite the phenotypic difference, MLL-r leukemias overlap in their gene expression profiles 39, suggesting a common mechanism for leukemogenesis. The molecular basis is that the majority (~75%) of the leukemia are caused by MLL-AF9/ENL, -AF4 and -ELL 9. These MLL fusion partners recruit other proteins (e.g., P-TEFb) and associate with each other to constitute SEC for transcription elongation, causing aberrant expression of MLL-target genes and leukemia initiation. Moreover, SEC was recruited by two other major fusions MLL-AF6 (5%) and -AF10 (5%) 11, 12. The second distinctive feature for MLL-r leukemias is DOT1L-mediated, genome-wide hyper-methylation of H3K79 24. DOT1L's catalytic activity is required for the aberrant gene expression and leukemia transformation. Pharmacological inhibition (e.g., by EPZ4777) further validated DOT1L is a drug target for MLL-r leukemia 25, 26.

The two leukemic gene regulatory pathways converge on the AHD domain of AF9/ENL, which binds to either DOT1L for H3K79 methylation or AF4/AFF4 for SEC formation 13,14. AF9/ENL AHD is therefore a potential drug target. However, to find a small-molecule inhibitor to disrupt these PPIs is challenging. Previous studies based on DOT1L-peptides yielded several 7-mer peptidomimetics with IC_50_s as low as 57 nM against AF9, while they are 3-10× less active against ENL 27. Neither cellular activities of these peptidomimetics nor their activities against AF9/ENL-AF4/AFF4 were reported. Their large molecular weights (> 800), possibly low cell permeability and metabolic stability due to their peptidic structures could be negative factors for biological studies and drug discovery.

### Compound 1 is a novel small-molecule inhibitor of AF9/ENL with non-competitive mode of inhibition

Three orthogonal biochemical methods, including Alpha, FP and Western-blot based pull-down assay, have confirmed that drug-like compound **1** inhibits the PPIs between AF9/ENL and DOT1L/AF4/AFF4 with IC_50_s of 0.9-3.5 µM, regardless of the large binding affinity differences of these PPIs (*K*_d_: 9-640 nM). Because both FP and Alpha assays are unsuitable for studies of AF9/ENL binding kinetics, we compared the IC_50_s of the inhibitor with the *K*_d_s of the PPIs (Table 1). DOT1L peptide is a competitive inhibitor. As shown in Figure S11A, our IC_50_ values of DOT1L against the AF9-DOT1L, -AF4 and -AFF4 interaction were found to increase proportionally with the decreasing *K*_d_s of the PPIs, following the Cheng-Prusoff equation and exhibiting a competitive mode of action. However, the corresponding IC_50_s of compound **1** do not increase with the decreasing *K*_d_ values, showing a non-competitive mode of inhibition. Similar trends were observed for ENL (Figure S11B). These results suggest compound **1** is a non-competitive inhibitor of AF9/ENL, while more investigation of the inhibitor-AF9/ENL-DOT1L interactions is needed.

### Compound 1 pharmacologically validates the AHD domain of AF9/ENL is a drug target

Co-IP showed that the inhibitor **1** dose-dependently reduced MLL-AF9-bound DOT1L and AF4 in Molm-13 leukemia cells (Figure 3A), indicating it is cell-permeable and can disrupt the target PPIs in cells. Such PPI inhibition resulted in significant reduction of DOT1L and its products H3K79me1/2, ENL and AFF4 in MLL-r leukemia cells (Figure 3B/C), which suppressed malignant gene expression related to MLL-AF9, DOT1L, HoxA9 and Myc (Figure 4), causing differentiation, apoptosis and inhibited proliferation of MLL-r leukemia and, more broadly, Myc-driven cancer cells. It is noteworthy that structurally similar, inactive compound **3** had no activities in these assays, largely excluding possible off-target effects. Moreover, consistent with ENL knockdown 31, compound **1**'s activity show AF9/ENL is required for MLL-r leukemia but is dispensable in solid tumor or normal tissues. Our results show that the AHD domain of AF9/ENL is a promising anticancer drug target and compound **1** represents a novel pharmacological lead.

### The AHD domain of AF9/ENL is required for recruitment of ENL and DOT1L-mediated H3K79 methylation

Compound **1** significantly reduced the nuclear levels of ENL, DOT1L and H3K79 methylation in MLL-AF9 and -AF4 leukemia cells (Figure 3B/C). Compound **1** also inhibited DOT1L from recruiting to MLL target genes HoxA9 and Myc for H3K79 methylation (Figure 4C). With respect to H3K79 methylation, compound **1** behaved similarly to EPZ4777 (Figure 3B and 4C). These results strongly support that recruitment of DOT1L and its mediated H3K79 methylation are dependent on the AF9/ENL-DOT1L interactions. This observation is of interest, particularly given DOT1L's N-terminal methyltransferase domain (1-472, which cannot binds to AF9/ENL) can tightly bind to nucleosomes and methylate H3K79 biochemically in our previous study 37. Rather, our results here show that DOT1L/H3K79 methylation in MLL-r leukemia cells is directed by AF9/ENL or more likely, AF9/ENL-containing SEC. Similarly, ENL is required for expression of MLL-target genes (e.g., HoxA9) and leukemogenesis 11, 31. Significant reduction of ENL by compound **1** shows recruiting ENL to chromatins is mainly dependent on its AHD domain.

In addition to AF4/AFF4 and DOT1L, AF9/ENL AHD has been found to interact with CBX8 (chromobox homolog 8), a protein in polycomb repressive complex 1 40, 41, as well as BCoR (BCL-6 corepressor) 42 with a similar binding mode 14. BCoR possesses the consensus sequence of LxVxIxLxxL and exhibits a high-affinity binding to AF9 AHD, while CBX8 with LxAxIxLxxI has a reduced affinity 14. The interactions between AF9/ENL and CBX8 or BCoR have been reported to be critical for MLL1-AF9/-ENL induced leukemic transformation 40, 41, 43, 44. Because of the similar binding mode, compound **1** could also inhibit these PPIs, which might contribute to the observed biological activities of the inhibitor.

## Conclusion

Compound **1** is a novel small-molecule inhibitor of the PPIs between AF9/ENL and DOT1L or AF4/AFF4. Such inhibition blocks the formation of SEC and recruitment of DOT1L to the MLL target genes with decreased H3K79 methylation. These molecular events significantly suppress malignant gene expression in MLL-r leukemia, inhibit cell proliferation, and promote differentiation and apoptosis. Compound **1** also exhibited strong antitumor activity in a mouse model of MLL-r leukemia without overt toxicity. It is a useful in vivo probe for biological studies as well as a pharmacological lead for further drug development.

## Methods

**Compound synthesis and characterization.** All chemicals for synthesis were purchased from Alfa Aesar (Ward Hill, MA) or Aldrich (Milwaukee, WI). The compound identity was characterized by ^1^H NMR on a Varian (Palo Alto, CA) 400-MR spectrometer. The purities of synthesized compounds were determined by a Shimadzu Prominence HPLC with a Zorbax C18 (or C8) column (4.6 x 250 mm) monitored by UV at 254 nm. The purities of the reported compounds were found to be > 95%. Synthesis and characterization of compounds **1**-**9** can be found in Supplemental Material.

**Plasmids and peptides.** cDNA for human AF9 AHD domain (475-568) and ENL AHD domain (489-559) was synthesized (by Genscript) and inserted into the pMAL c5X expression plasmid. The following peptides were purchased from Genscript:

DOT1L: Biotin-AHX-NKLPVSIPLASVVLPSRAERARST (for Alpha assay and pull-down assay) and FITC-AHX-NKLPVSIPLASVVLPSRAERARST (for FP assay);

AF4: Biotin-AHX-QSLMVKITLDLLSRIPQPPGK (for Alpha assay) and FITC-AHX-QSLMVKITLDLLSRIPQPPGK (for FP assay)

AFF4: Biotin-AHX-YPLIVKIDLNLLTRIPGKPYK (for Alpha assay) and FITC-AHX-YPLIVKIDLNLLTRIPGKPYK (for FP assay)

**Protein expression and purification.** The expression plasmids were used to transform *E. coli* BL21(DE3) strain (Novagen, USA) and protein expression was induced in the presence of 0.4 mM isopropyl β-D-1-thiogalactopyranoside (IPTG) at 16 °C overnight. Cells were collected and lysed using a French press (GlenMills) in a lysis buffer (50 mM HEPES, 200 mM NaCl, 1 mM DTT, pH 7.4). Upon centrifugation, the supernatant was applied to an amylose resin column (GE Healthcare) and the recombinant protein was eluted with 20 mM HEPES, 10 mM Maltose, pH 7.4, which was further purified to be > 95% (SDS-PAGE) using a size exclusion column (HiLoad 16/60 Superdex 200, GE Healthcare).

**Alpha assays.** Alpha assays were developed using Perkin-Elmer AlphaLisa anti-MBP kit, which contains streptavidin donor beads and anti-MBP acceptor beads. The assay was performed using a MBP-protein (5 nM), a biotinylated peptide (40 nM), and increasing concentrations of a compound in 25 µL buffer (PBS with 0.5 % BSA, pH 7.5) in 384-well plates according to the manufacturer's protocol and measured with a Tecan SPARK microplate reader. Data were imported into Prism (version 5.0), and IC_50_ values from 3 independent experiments with standard deviation were obtained by using a standard dose-response curve fitting.

**Fluorescence polarization assay.** It was performed in 96-well black microplates with each well containing a Fluorescein-labeled peptide (2 nM) and increasing concentration of a AF9/ENL protein in a buffer (20 mM HEPES, pH 7.5, 0.01% Triton X-100, 100 µL) for *K*_d_ determination. For IC_50_ determination, a Fluorescein-labeled peptide (2 nM), AF9/ENL protein (100 nM) and increasing concentration of a compound in the same buffer were placed in each well. Upon incubation of 1h, the FP value of each well was determined using a Tecan SPARK microplate reader. Data were similarly processed to obtain *K*_d_ or IC_50_ values.

**Western blot based pull-down assay.** Biotinylated DOT1L peptide (0.25 mg/mL) was incubated with streptavidin agarose beads in PBS containing 0.1% Triton X-100 (400 µL) for 3h to yield, after washing, DOT1L-coated beads. MBP-AF9 AHD (0.2 µM, 40 µL) was pre-incubated with a compound for 30 min before adding to the beads. After 6 hours incubation at 4 °C, the beads were collected, thoroughly washed, and subjected to SDS-PAGE and analyzed by Western blot using an MBP Mouse mAb (#2396, Cell signaling).

**Inhibition of DOT1L, LSD1 and p300** was performed using our previously reported methods 29, 37, 38.

**Co-immunoprecipitation.** 10^8^ Molm-13 cells were treated with a compound or DMSO for 48 hr. Co-immunoprecipitation of the nuclear protein extracts (450 µg of total protein from 10^8^ molm-13 cells) was performed using a Nuclear Complex Co-IP Kit (#54001, Active Motif). Briefly, 3 µg of MLL-N Rabbit mAb (#14689, Cell signaling) and 100 µL Protein G Agarose Beads (#37478, Cell signaling) were added and incubated with the nuclear extractions overnight at 4 °C. The Agarose beads with captured antibody and relevant protein complexes were isolated, washed and subjected to Western blot for protein detection.

**Western blot.** 3x10^6^ cells/well were treated with increasing concentrations of a compound for 3 days and nuclear proteins extracted with the EpiQuik Nuclear Extraction Kit (Epigentek) according to the manufacturer's protocol. Equal amounts of proteins were separated on SDS-PAGE and transferred to PVDF membranes. The blots were probed with primary antibodies, followed by anti-rabbit or anti-mouse IgG (Thermo Scientific) secondary antibodies. The primary antibodies used in this research were MBP Mouse mAb (#2396, Cell signaling), DOT1L Rabbit mAb (#77087, Cell signaling), MLL-N Rabbit mAb (#14689, Cell signaling), ENL Rabbit mAb (#14893, Cell signaling), Di-Methyl-Histone H3K79 Rabbit mAb (#5427, Cell signaling), Mono-Methyl-Histone H3K79 Rabbit mAb (#9398, Cell signaling), Histone H3 Rabbit mAb (#4499, Cell signaling), AF4 Rabbit pAb (#NBP1-28705, Novusbio) and AFF4 Rabbit pAb (#ab103586, Abcam).

**Antiproliferation assay.** Proliferation inhibition assays for suspension blood cancer cells were performed using an XTT assay kit (Roche). Proliferation inhibition assays for solid tumor cell lines MCF-7, MDA-MB-231, Panc1, Panc28 were performed using an MTT assay (Sigma). The antiproliferation EC_50_ values were determined using Prism 5 and the reported results were the mean values of at least three independent experiments. The incubation time for compound 1 was 7 days and that for EPZ4777 was 14 days.

**Cytospin/Wrigth-Giemsa staining.** The MV4;11 and Molm-13 cells were plated in 6-well plates (2.5 mL/well) at an initial concentration of 10^5^ cells/mL and treated with a compound or DMSO at 37 °C in a 5% CO2 incubator for 5 days. Cytospins were performed to smear cells (100 µL, 10^6^ cells/mL) on slide. After air dry, the slides were Wright's-Giemsa stained and air-dried for over 30 mins before examination.

**Flow cytometry.** For Annexin V apoptosis assay, 10^5^ cells/ml were incubated with increasing concentrations of a compound for 5 days. Apoptosis was determined using the FITC Annexin V Apoptosis Detection Kit I (BD Bioscience) using the manufacturer's protocol. For other FACS assays, cells were labeled with fluorochrome-conjugated monoclonal antibodies against human CD14 and CD11b (BD Biosciences) according to the manufacturer's recommendation. Cells were analyzed using a FACS Calibur (BD Biosciences/Applied Biosystems) and data were processed using the program Flowjo (version7.6.5).

**RNA extraction and Quantitative real-time PCR (qPCR).** 10^5^ cells/ml were incubated with a compound for 4 days and the RNA was extracted using RNeasy mini kit (#74104, Qiagen). 100-1000 ng of total RNA was reverse transcribed using iScript™ Reverse Transcription Supermix (Bio-Rad) using the manufacturer's protocol. Quantitative real-time PCR was carried out using Fast SYBR Green Master Mix (Applied Biosystems) according to the manufacturer's instructions. Measurements were performed in triplicate, using GAPDH as the reference gene. Real-time PCR was performed using Biosystems Step One Plus detection system. The following sequences of primers are used:

MYC (forward: 5'-CACCGAGTCGTAGTCGAGGT-3'; reverse: 5'-TTTCGGGTAGTGGAAAACCA-3');

HoxA9 (forward: 5'-TACGTGGACTCGTTCCTGCT-3'; reverse: 5'-CGTCGCCTTGGACTGGAAG-3');

Meis1 (forward: 5'-CCAGCATCTAACACACCCTTAC-3'; reverse: 5'-TATGTTGCTGACCGTCCATTAC -3');

GAPDH (forward: 5'-GCGAGATCCCTCCAAAATCAA-3'; reverse: 5'-GTTCACACCCATGACGAACAT -3')

**Chromatin immunoprecipitation.** Upon treatment with Cpd-1 for 3 days, 10^7^ Molm-13 cells were cross-linked with 1% formaldehyde at room temperature for 10 min, followed by the addition of 125 mM glycine. Cells were lysed with nuclear lysis buffer and sonicated to ~100-1000 bp fragments, which was incubated at 4 °C overnight with the H3K79me2 antibody (#5427, Cell signaling) and IgG (C15410206, Diagenode). Protein A/G Magnetic Beads (10 µL, Novus Biologicals) were added and incubated for 2 h. The beads were washed 3x with RIPA buffer and 2x with TE buffer. DNA on the beads was eluted for 2 hours at 68 °C in 100 μL of an elution buffer (20 mM Tris pH 7.5, 5 mM EDTA, 50 mM NaCl, 1% SDS, 50 μg/ml proteinase K) (2x), and purified using a ChIP DNA Clean & Concentrator kit (Novus Biologicals). qPCR was done using the method described above.

**Library Preparation.** The RNA integrity for each sample was assessed with a RNA 6000 Nano chip on a 2100 Bioanalyzer (Agilent; Santa Clara, CA). The average RNA integrity number (RIN) for the sample set was 9.68. In total, 3 μg RNA per sample was used as the input for RNA sample preparation. Sequencing libraries were generated using the NEBNext® Ultra™ RNA Library Prep Kit for Illumina® (NEB) following the manufacturer's recommendations and index codes were added to attribute sequences to each sample. Briefly, mRNA was purified from total RNA using poly-T oligo-attached magnetic beads. Fragmentation was performed using divalent cations under elevated temperature in NEBNext First Strand Synthesis Reaction Buffer (5X). First strand cDNA was synthesized using random hexamer primers and M-MuLV Reverse Transcriptase (RNase H). Second strand cDNA synthesis was subsequently performed using DNA Polymerase I and RNase H. The remaining overhangs were converted into blunt ends using exonuclease/polymerase activity. After adenylation of the 3' ends of DNA fragments, NEBNext Adaptor with hairpin loop structure was ligated to prepare the samples for hybridization. In order to select cDNA fragments of preferentially 150~200 bp, the library fragments were purified using the AMPure XP system (Beckman Coulter). Next, 3 μl USER Enzyme (NEB) was incubated with size-selected, adaptor-ligated cDNA for 15 min at 37 °C followed by 5 min at 95 °C before PCR. PCR was then performed with Phusion High-Fidelity DNA polymerase, Universal PCR primers, and the Index (X) Primer. Finally, the PCR products were purified (AMPure XP system). Final library quality control was carried out by measuring the fragment size on a DNA1000 chip on a 2100 BioAnalyzer (Agilent).

**Clustering and Sequencing.** Cluster generation of the denatured libraries was performed utilizing the HiSeq X PE Cluster Kit V2.5 (Illumina) according to the manufacturer's instructions. Sequencing was performed on a Novaseq6000 sequencer (Illumina) using S4 flowcell with paired-end 101 bp reads and a 6 bp index read culminating in an average output of 45 million paired-end reads per sample. Sequence read data were processed and converted to FASTQ format by Illumina BaseSpace analysis software (v2.0.13).

**Bioinformatics Analysis.** The pair-ended reads were mapped to the human genome (UCSC hg19) using software STAR (https://github.com/alexdobin/STAR) with NCBI RefSeq genes as the reference. The gene-based read counts generated by STAR were used as the measurement for gene expression. R Bioconductor package DESeq2 (http://bioconductor.org/) was used to analyze the gene-based read counts to detect differentially expressed genes between the groups of interest. The false discovery rate (FDR) of the differentially expressed genes was estimated using Benjamini and Hochberg method. FDR < 0.05 was considered statistically significant. Gene set enrichment analysis (GSEA) was performed using the GSEA software (https://www.gsea-msigdb.org/gsea/index.jsp).

**In vivo antitumor studies.** All of the mouse studies were conducted in strict compliance with an IRB-approved protocol. NOD-SCID mice (4 to 6 weeks old, from Jackson lab) were obtained and maintained under specific pathogen-free conditions. 10^7^ MV4;11 cells in medical grade saline and Matrigel (1:1, 0.1 mL) were inoculated subcutaneously and palpable tumors (2-3 mm in diameter) were developed in ~ 2 weeks. Mice were treated with compound **1** (15 mg/kg/day for 10 days) in saline (0.1 mL) administered intraperitoneally. Tumors were measured every 2 days and estimated by using the formula a×b^2^/2.

**Statistical analysis.** At least three independent experiments were carried out to generate each dataset. The significance of experimental differences was evaluated by use of the Student's *t* test (Prism 5.0). Results are expressed as mean ± SEM.

**Data sharing.** RNA-seq data has been deposited to GEO with accession code GSE151660.

## Supplementary Material

Supplementary figures, table, materials and methods.

## Figures and Tables

**Table 1:** Biochemical binding affinity (*K*_d_) and inhibition of PPIs.

PPI	*K*_d_ (µM) ^a^	DOT1L IC_50_ (µM) ^b^	Cpd-1 IC_50_ (µM) ^b^
AF9-DOT1L	0.087 ± 0.009	0.013 ± 0.001	3.5 ± 0.2
AF9-AF4	0.018 ± 0.001	0.060 ± 0.012	1.5 ± 0.2
AF9-AFF4	0.015 ± 0.001	0.095 ± 0.016	2.5 ± 0.1
ENL-DOT1L	0.64 ± 0.06	n.d.^c^	n.d.^c^
ENL-AF4	0.0096 ± 0.0017	13.2 ± 1.1	0.94 ± 0.09
ENL-AFF4	0.031 ± 0.006	2.0 ± 0.1	3.3 ± 0.3

^a^Using FP assay; ^b^Using Alpha assay; ^c^Not determined.

## Abbreviations

ALL: acute lymphocytic leukemia
Alpha: amplified luminescent proximity homogeneous assay
AML: acute myeloid leukemia
ChIP: chromatin immunoprecipitation
FP: fluorescence polarization
GSEA: gene set enrichment analysis
H3K79: histone H3 lysine 79
H3K79-Me1 or -Me2: mono- or di-methylated H3K79
MBP: maltose-binding protein
MLL: mixed lineage leukemia
MLL-r: MLL-rearranged
PPI: Protein-protein interactions
SEC: super elongation complexes
