# Supplementary material for: Small-molecule inhibitor of AF9/ENL-DOT1L/AF4/AFF4 interactions suppresses malignant gene expression and tumor growth
Source: Theranostics. 2021 Jul 13;11(17):8172–84. doi: 10.7150/thno.56737 (PMC8344022; doi:10.7150/thno.56737)
Supplement: Supplementary file 1 — Supplementary figures, table, materials and methods. [file thnov11p8172s1.pdf]

Supplementary Materials

Small-molecule inhibitor of AF9/ENL-DOT1L/AF4/AFF4 interactions suppresses malignant gene expression and tumor growth

Fangrui Wu,[1,2,†] Shenyou Nie,[1,†] Yuan Yao,[1,†] Tong Huo,[1,†] Xin Li,[1] Xiaowei Wu,[1] Jidong Zhao,[1] Yi-Lun Lin,[1] Yinjie Zhang,[1] Qianxing Mo,[3] and Yongcheng Song[1,2,*]

[1]Department of Pharmacology and Chemical Biology, [2]Dan L. Duncan Comprehensive Cancer Center, Baylor College of Medicine, 1 Baylor Plaza, Houston, TX 77030, USA.

[3]H. Lee Moffitt Cancer Center & Research Institute, 12902 USF Magnolia Drive, Tampa, FL 33612, USA.

Table of Contents:

Figure S1-S9 Page S2-S10

Table S1 Page S11

Material and methods Page S12

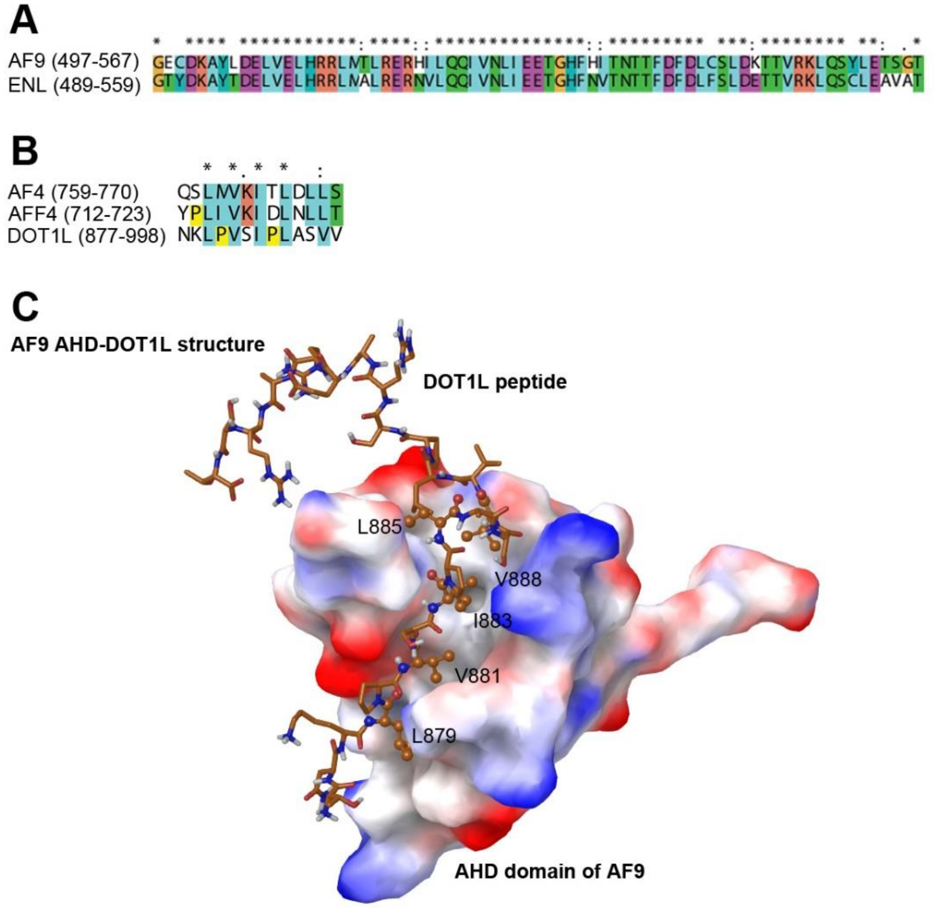

Figure S1. Interactions between the AHD domain of AF9/ENL and AF4/AFF4/DOT1L. (**A**) Sequence alignment of the AHD domains of AF9 and ENL, showing 82% identity and 89% similarity; (**B**) Sequence alignment of AF4, AFF4 and DOT1L peptide with a consensus sequence of LxVxIxLxxL/V, which bind to AF9/ENL AHD; (**C**) NMR structure (PDB: 2MV7) of the AF9 AHD-DOT1L complex, with AF9 AHD shown as an electrostatic surface and the consensus interacting residues of DOT1L as ball and stick models.

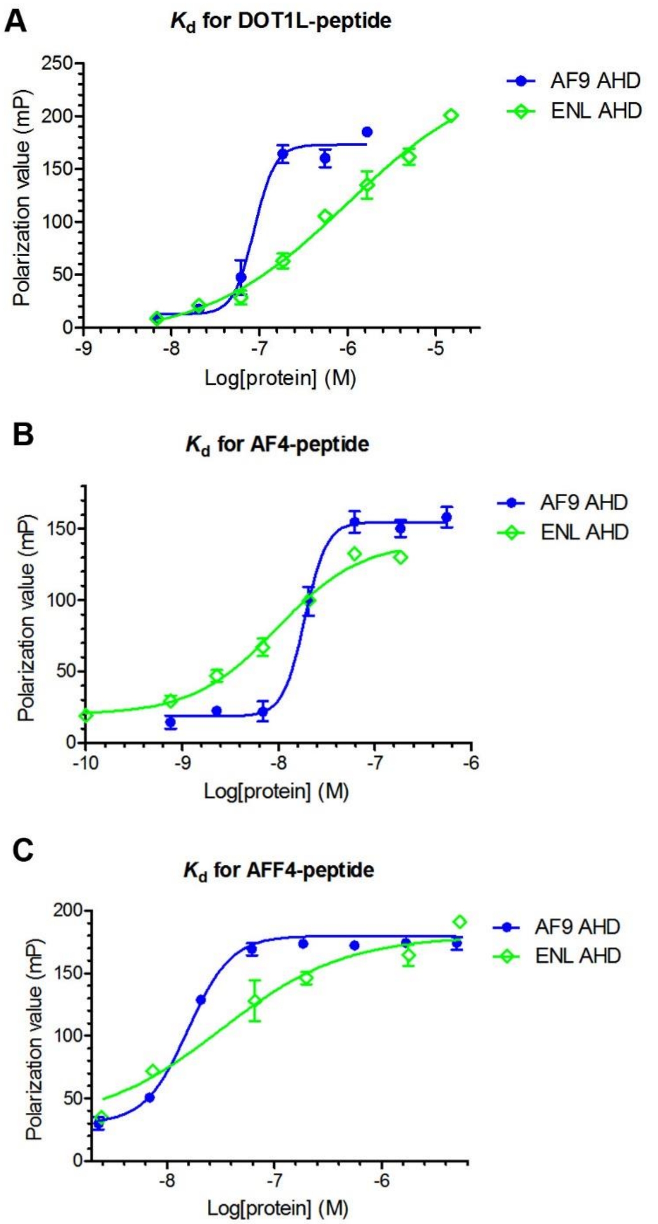

Figure S2. Dose-responsive curves of FP assays from which the K_d values between AF9/ENL and (**A**) DOT1L peptide; (**B**) AF4 peptide; and (**C**) AFF4 peptide were determined.

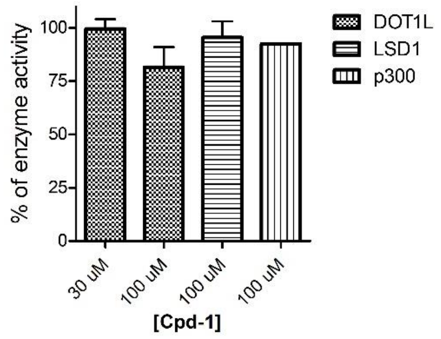

Figure S3. Compound **1** did not significantly inhibit the enzyme activities of DOT1L (the left two columns), LSD1, and p300 histone acetyltransferase at 30 or 100 μM, using our previous reported methods [Ref. 36, 37, and 44].

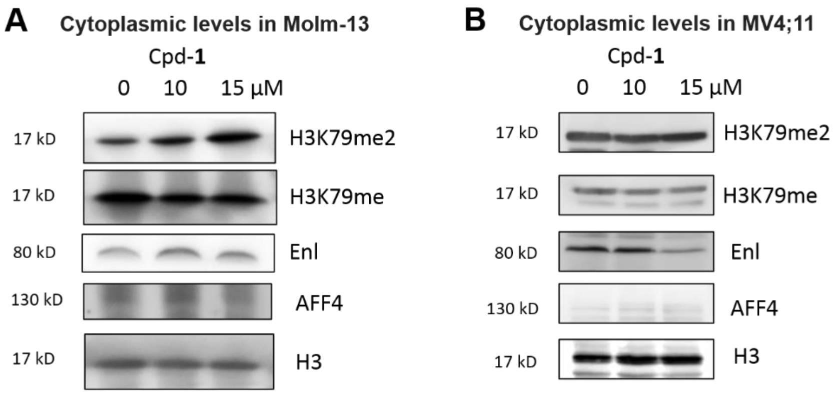

Figure S4. The cytoplasmic protein levels after treatment with Cpd-**1** for 3 days. (A) In contrast to the protein levels in the nucleus, Cpd-**1** did not significantly reduce the cytoplasmic levels of H3K79me1 and me2, ENL and AFF4 in Molm-13 cells; (**B**) Cpd-**1** did not significantly reduce the cytoplasmic protein levels of H3K79me1 and me2, ENL and AFF4 in MV4;11 cells, except for ENL at 15 µM.

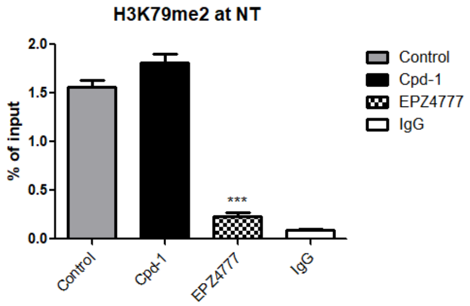

Figure S5. Treatment with Cpd-**1** (5 μM for 4 days) did not reduce the H3K79-Me2 level in a non-transcribed DNA region (Ref. 32) in Molm-13 cells, presumably because SEC is not recruited there. In contrast, DOT1L inhibitor EPZ4777 decreased H3K79-Me2 in this DNA region.

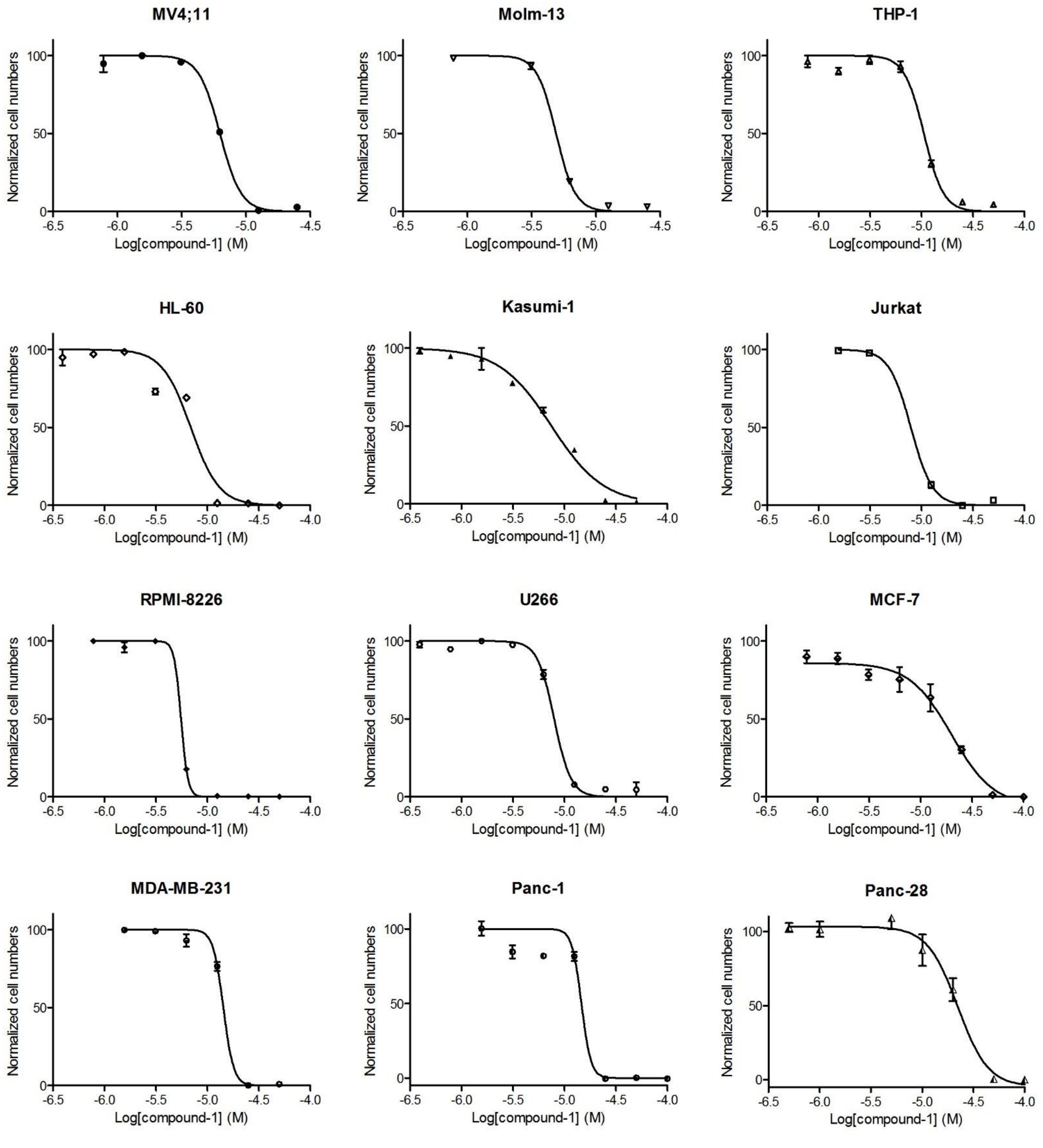

Figure S6. Dose-responsive curves for the activities of compound **1** (7-day incubation) against proliferation of cancer cell lines.

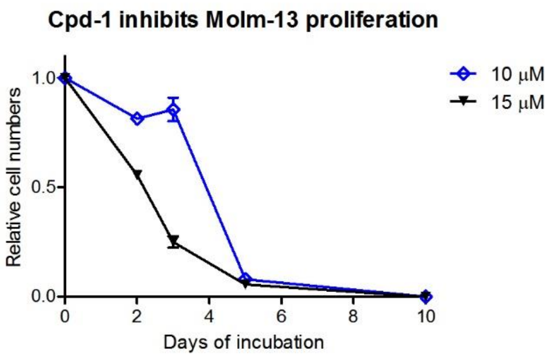

Figure S7. Time-dependent antiproliferative activity of compound **1** against Molm-13 cells, showing at 10 µM (~2x EC_{50}) it did not significantly inhibit the cell proliferation on Day-2 or 3, but showed >90% inhibition on Day-5. At 15 µM (~3x EC_{50}) it had more potent antiproliferative activity.

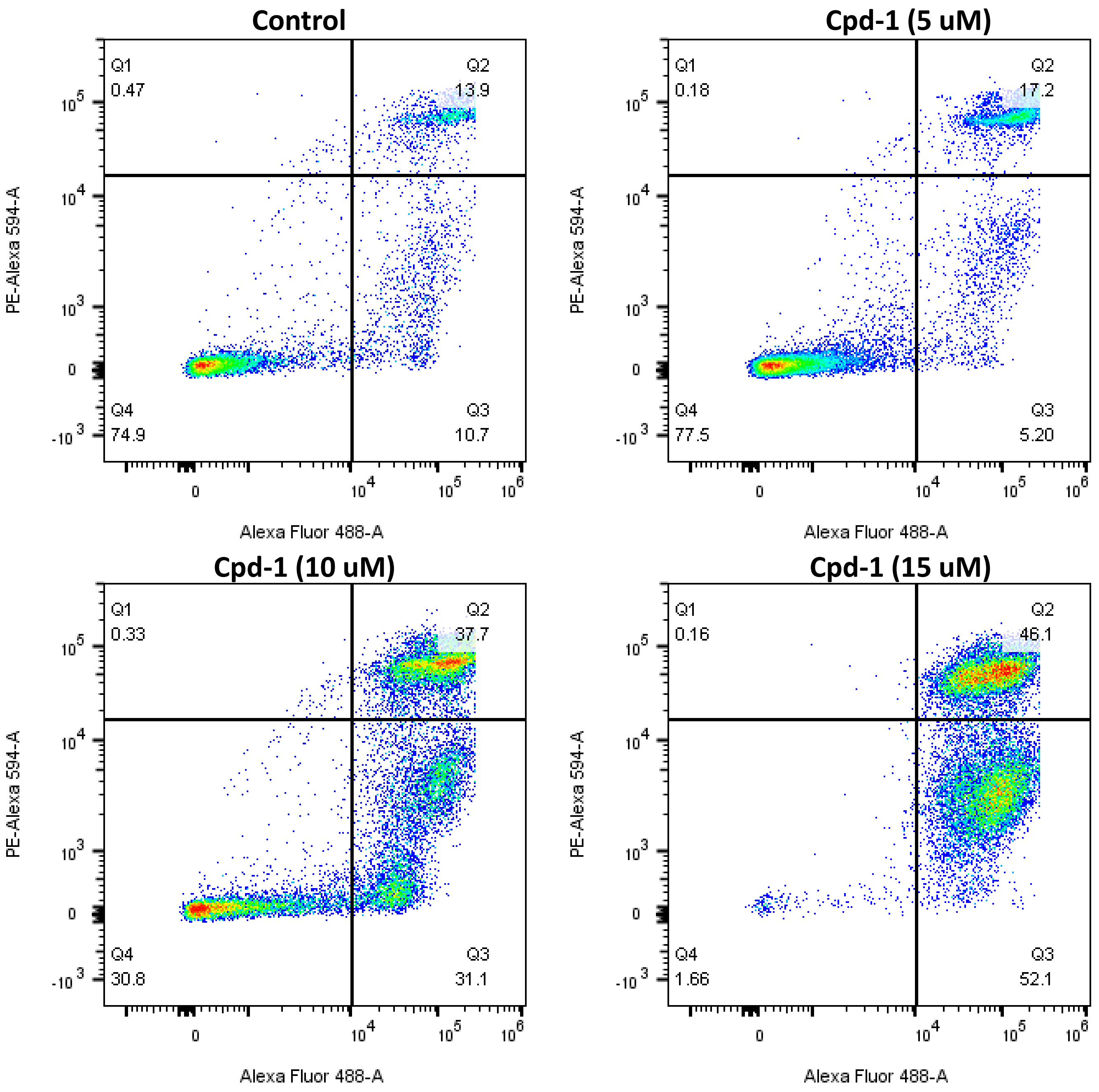

Figure S8. Compound **1** induced significant apoptosis of Molm-13 leukemia cells at 10 and 15 µM (5-day incubation), as compared to the control. 5 µM of **1** did not cause significant apoptosis. The upper right (Q2) number in each figure refers to the proportion of propidium iodide-positive, apoptosed cells and the lower right (Q3) number refers to that of annexin-V-positive cells (early apoptosis).

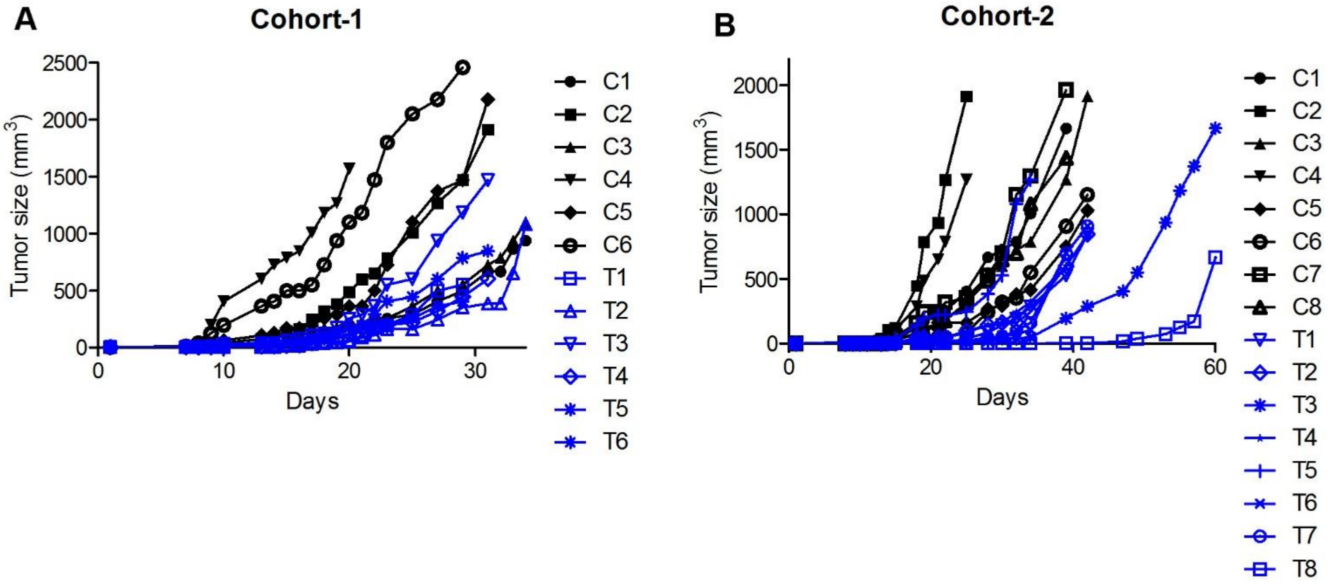

Figure S9. MV4;11 tumor growth curves for individual mice for (**A**) the cohort-1 and (**B**) cohort-2 experiments, with control mice shown in black and treated mice in blue.

Cohort-1: Cohort-2:

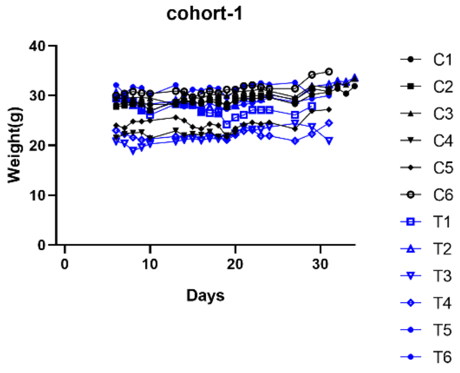

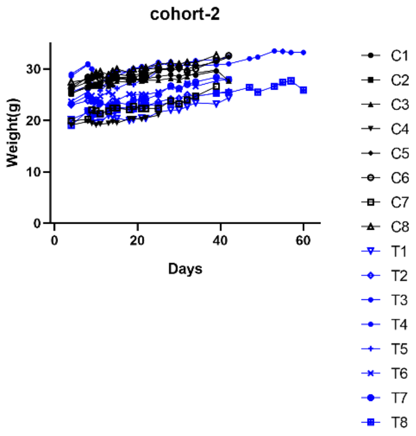

Figure S10. Treatment with Cpd-**1** did not significantly affect the weights of experimental mice, showing no obvious toxicities.

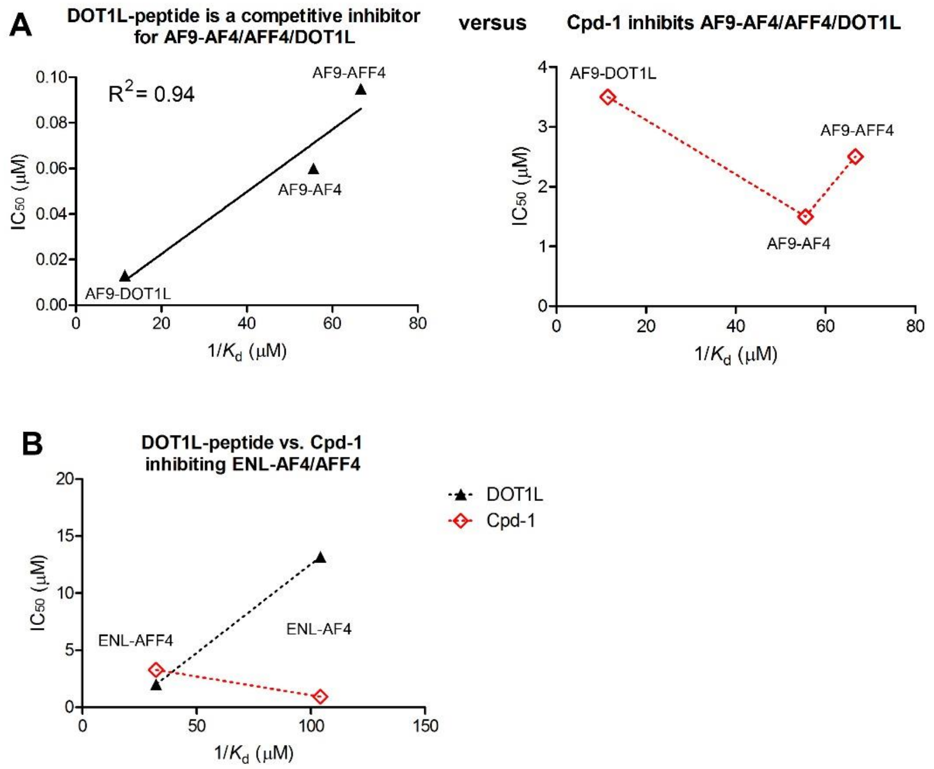

Figure S11. Compound **1** is likely a non-competitive inhibitor of the PPIs between AF9/ENL and AF4/AFF4/DOT1L. All data are from Table 1. (**A**) Plots of IC_{50} vs. $1/K_d$ for non-biotinylated DOT1L peptide and Cpd-**1** inhibiting AF9 AHD. DOT1L-peptide is a competitive inhibitor of AF9 AHD, with its IC_{50} values against the AF9-DOT1L, -AF4 and -AFF4 interaction proportionally increase with the decreasing K_d values of these PPIs, following the Cheng-Prusoff equation $IC_{50} = K_i + K_i \times [L]/K_d$, where K_i is the inhibition constant of the inhibitor and [L] is the concentration of the biotinylated peptide (40 nM). The IC_{50} values of compound **1** do not increase with the decreasing K_d values of the PPIs, showing a non-competitive mode of inhibition; (**B**) Plots of IC_{50} vs. $1/K_d$ for non-biotinylated DOT1L peptide and Cpd-**1** inhibiting ENL AHD. Similarly, competitive inhibitor DOT1L's IC_{50} values increase with the decreasing K_d values of the ENL-AFF4 and -AF4 interactions, but not those of compound **1**.

Table S1. Cytotoxicity EC_{50} (μM) of compounds **1**, **3** (7-day incubation) and EPZ4777 (14-day).

	Cpd-**1**	Cpd-**3**	EPZ4777
MV4;11	4.7 ± 0.4	>50	1.1 ± 0.3
Molm-13	4.9 ± 0.1	>50	1.2 ± 0.2
THP-1	11.4 ± 0.6	>50	3.4 (Ref 25)
HL60	7.3 ± 0.1	>50	>50 (Ref 25)
Kasumi-1	8.9 ± 0.3	>50	33 (Ref 25)
Jurkat	9.7 ± 0.3	>50	>50 (Ref 25)
RPMI-8226	3.3 ± 0.1	>50	>50
U266	7.9 ± 0.4	>50	>50
MCF-7	19.8 ± 0.3	>50	>50
MDA-MB-231	13.5 ± 0.2	>50	>50
Panc-1	15.1 ± 0.2	>50	>50
Panc-28	23.8 ± 0.2	>50	>50

Synthesis of inhibitors

All chemicals for synthesis were purchased from Alfa Aesar (Ward Hill, MA), Aldrich (Milwaukee, WI) or Combi-Blcoks (San Diego, CA). The identity of the synthesized compounds was characterized by ^{1}H and ^{13}C NMR on a Varian (Palo Alto, CA) 400-MR spectrometer and mass spectrometer (Shimadzu LCMS-2020). The identity of compound **1** was confirmed with high resolution mass spectra (HRMS) using an Agilent 6550 iFunnel quadrupole-time-of-flight (Q-TOF) mass spectrometer with electrospray ionization (ESI). The purities of the final compounds were determined to be >95% with a Shimadzu Prominence HPLC using a Zorbax C18 (or C8) column (4.6 x 250 mm) monitored by UV at 254 nm. The synthetic methods for compounds **1-9** are shown in Schemes 1, 2 and 3.

Scheme 1. General Methods for Synthesizing Compounds **1, 7, 8** and **9**[a]**.**

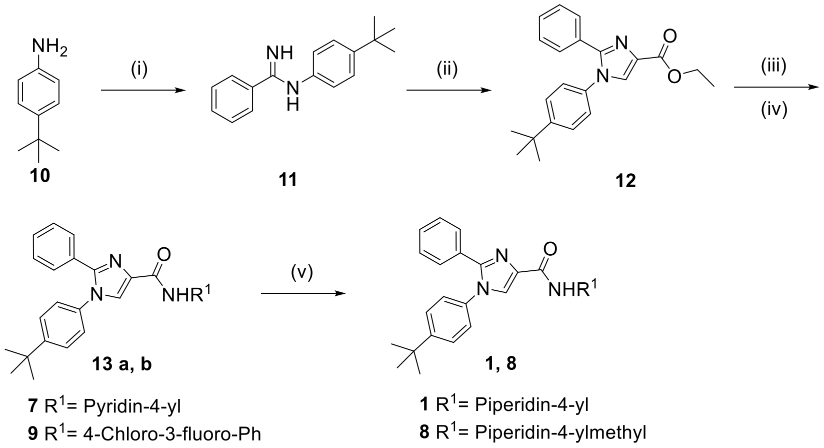

[a]*Reagents and conditions:* (i) Benzonitrile, NaH, DMSO, 0 °C to rt, 12 h, 76%; (ii) Ethyl bromopyruvate, CBr_4, MeCN, 90 °C, 12 h, 50%; (iii) NaOH, THF-H_2O(v/v, 3/1), reflux, 5 h; (iv) R^1NH_2, HATU, DIPEA, DMF, 12 h, 60-66% for 2 steps; (v) HCl (4 N in *p*-dioxane), CH_2Cl_2, rt, 5 h, 90-95%.

To a solution of 4-*tert*-butylaniline (**10**, 7.5 g, 50 mmol) in DMSO (50 mL) was slowly added NaH (2.4 g, 60 mmol, 60% dispersion in mineral oil) at 0 °C. After stirring at 0 °C for 1 h, benzonitrile (5.69 g, 55 mmol) in DMSO (30 mL) was added. The resulting mixture was warmed to room temperature and stirred for another 12 h. The reaction was quenched with icy water and extracted with ethyl acetate (3 × 100 mL) and the combined organic

layers were washed with water and brine and dried over Na_2SO_4. The volatiles were removed *in vacuo* to afford a brown solid, which was purified by recrystallization (hexanes/ethyl acetate) to afford N-(4-(tert-butyl)phenyl)benzimidamide (**11**, 9.62 g, 76%) as a white solid. ^{1}H NMR (400 MHz, $CDCl_3$) δ 7.85 (d, *J* = 7.0 Hz, 2H), 7.49 – 7.39 (m, 3H), 7.37 (d, *J* = 8.3 Hz, 2H), 6.92 (d, *J* = 8.3 Hz, 2H), 4.91 (s, 2H), 1.34 (s, 9H); ^{13}C NMR (100 MHz, $CDCl_3$) δ 155.1, 146.7, 145.8, 136.0, 130.5, 128.6, 126.9, 126.4, 121.2, 34.4, 31.6.

To a solution of compound **11** (8 g, 62 mmol) in acetonitrile (60 mL) was added ethyl bromopyruvate (4.2 g, 68 mmol) and tetrabromomethane (8.29 g, 25.0 mmol). After stirring at room temperature for 12 h, the solvent was removed under vacuum. The mixture was extracted with ethyl acetate (3 × 100 mL) and the combined organic layers were washed with water and brine and dried over Na_2SO_4. The volatiles were removed *in vacuo* to afford a crude oil, which was purified by column chromatography (silica gel, hexanes: ethyl acetate from 5:1 to 3:1) to afford ethyl 1-(4-(tert-butyl)phenyl)-2-phenyl-1H-imidazole-4-carboxylate **12** (2.92, g, 50%) as colorless oil. ^{1}H NMR (400 MHz, CDCl3) δ 7.72 (s, 1H), 7.32 (t, *J* = 8.3 Hz, 4H), 7.19 – 7.10 (m, 3H), 7.03 (d, *J* = 8.4 Hz, 2H), 4.31 (dd, *J* = 14.2, 7.1 Hz, 2H), 1.29 (t, *J* = 7.1 Hz, 3H), 1.23 (s, 9H); ^{13}C NMR (100 MHz, CDCl3) δ 162.8, 151.9, 147.5, 134.8, 133.1, 129.1, 128.84, 128.79, 128.4, 127.9, 126.4, 125.0, 60.4, 34.6, 31.1, 14.3.

To a solution of compound **12** (2.2 g, 6.3 mmol) in THF/H_2O (9/3 mL) was added sodium hydroxide (632 mg, 15.8 mmol). The resulting mixture was refluxed for 5 h. The solvent was removed under vacuum and acidified with 3N HCl, extracted with ethyl acetate (3 × 50 mL) and the combined organic layers were washed with water and brine and dried over Na_2SO_4. The volatiles were removed *in vacuo* to afford a crude product, which was used for the next step without further purification.

To a solution of the crude acid and 4-amino-1-Boc-piperidine (1.51 g, 7.56 mmol) in DMF (30 mL) was added N, N-diisopropylethylamine(2.19 mL, 12.6 mmol) and 1-[Bis(dimethylamino)methylene]-1H-1,2,3-triazolo[4,5-b]pyridinium 3-oxide hexafluorophosphate (HATU, 3.59 g, 9.45 mmol). The mixture was stirred for 12 h before it was quenched with H_2O. The mixture was extracted with ethyl acetate (3 × 80 mL) and the combined organic layers were washed with water and brine and dried over Na_2SO_4. The volatiles were removed *in vacuo* to

afford a crude oil, which was purified by column chromatography (silica gel, hexanes: ethyl acetate from 2:1 to 1:1) to afford **13** (2.1 g, 66% for 2 steps) as colorless oil. **13a** (Intermediate of compound **1**): ^{1}H NMR (400 MHz, $CDCl_3$) δ 7.78 (s, 1H), 7.45 – 7.38 (m, 4H), 7.35 – 7.28 (m, 3H), 7.19 (d, *J* = 8.3 Hz, 1H), 7.13 (d, *J* = 8.5 Hz, 2H), 4.21 – 4.06 (m, 3H), 2.99 – 2.89 (m, 2H), 2.06 – 1.99 (m, 2H), 1.54 – 1.44 (m, 11H), 1.36 (s, 9H); ^{13}C NMR (100 MHz, $CDCl_3$) δ 161.7, 154.5, 152.0, 146.3, 136.6, 135.2, 129.7, 128.7, 128.6, 128.1, 126.3, 125.2, 125.01, 124.98, 79.2, 46.2, 42.7, 34.6, 32.0, 31.1, 28.3.

Compound 7: 1-(4-(Tert-butyl)phenyl)-2-phenyl-N-(pyridin-4-yl)-1H-imidazole-4-carboxamide

^{1}H NMR (400 MHz, DMSO-d_6) δ 11.75 (s, 1H), 8.76 (d, *J* = 7.0 Hz, 2H), 8.70 (s, 1H), 8.53 (d, *J* = 7.1 Hz, 2H), 7.51 (d, *J* = 8.6 Hz, 2H), 7.46 – 7.35 (m, 5H), 7.31 (d, *J* = 8.5 Hz, 2H), 1.29 (s, 9H); ^{13}C NMR (100 MHz, DMSO-d_6) δ 161.5, 153.4, 151.8, 146.8, 141.8, 134.3, 133.8, 129.6, 129.3, 129.0, 128.5, 128.4, 126.4, 125.6, 115.1, 34.6, 31.0; MS (ESI) $[M+H]^+$ 397.2.

Compound 9: 1-(4-(Tert-butyl)phenyl)-N-(4-chloro-3-fluorophenyl)-2-phenyl-1H-imidazole-4-carboxamide

^{1}H NMR (400 MHz, $CDCl_3$) δ 9.13 (s, 1H), 7.95 (dd, *J* = 6.5, 2.5 Hz, 1H), 7.84 (s, 1H), 7.56 – 7.50 (m, 1H), 7.45 – 7.38 (m, 4H), 7.36 – 7.27 (m, 3H), 7.17 – 7.08 (m, 3H), 1.35 (s, 9H); ^{13}C NMR (100 MHz, DMSO-d_6) δ 161.1, 154.8, 152.4, 151.8, 146.6, 136.6, 136.1, 135.2, 129.9, 129.5, 129.2, 128.7, 127.3, 126.8, 126.0, 122.0, 120.9, 120.9, 119.4, 119.2, 117.2, 117.0, 35.0, 31.4; MS (ESI) $[M+H]^+$ 448.2.

To a solution of compound **13a** (1.5 g, 2.98 mmol) in dicholromethane (20 mL) was added dropwise HCl (2.2 mL, 4 N in *p*-dioxane) at 0 °C. The reaction mixture was warmed to room temperature and stirred for 5 h. The volatiles were removed *in vacuo* to afford an oil, which was triturated in diether ether and solidified to give the final product as a hydrochloric salt (**1**, 1.18 g, 90%) as a white powder.

Compound 1: 1-(4-(tert-butyl)phenyl)-2-phenyl-N-(piperidin-4-yl)-1H-imidazole-4-carboxamide hydrochloride

^{1}H NMR (400 MHz, DMSO-d_6) δ 9.43 (d, *J* = 8.9 Hz, 1H), 9.23 (d, *J* = 6.3 Hz, 1H), 9.10 (d, *J* = 8.7 Hz, 1H), 8.71 (s, 1H), 7.55 – 7.50 (m, 2H), 7.50 – 7.38 (m, 5H), 7.38 – 7.32 (m, 2H), 4.11 (s, 1H), 3.33 (d, *J* = 11.6 Hz,

2H), 3.00 (d, J = 10.6 Hz, 2H), 2.03 – 1.85 (m, 4H), 1.28 (s, 9H); ^{13}C NMR (100 MHz, DMSO-d_6) δ 157.9, 152.5, 145.4, 133.3, 131.0, 130.2, 129.7, 128.5, 126.6, 126.5, 125.7, 124.6, 44.1, 41.8, 34.6, 31.0, 28.0; HRMS (ESI$^+$) calcd for $C_{25}H_{30}N_4O$ [M+H]$^+$ 403.2498, found 403.2491.

Compound 8: 1-(4-(Tert-butyl)phenyl)-2-phenyl-N-(piperidin-4-ylmethyl)-1H-imidazole-4-carboxamide hydrochloride

^{1}H NMR (400 MHz, DMSO-d_6) δ 9.01 (s, 1H), 8.70 (s, 2H), 8.21 (s, 1H), 7.50 (dd, J = 8.5, 3.0 Hz, 2H), 7.43 – 7.34 (m, 5H), 7.30 (dd, J = 8.5, 2.7 Hz, 2H), 3.27 – 3.18 (m, 4H), 2.81 (q, J = 11.5 Hz, 2H), 1.89 – 1.76 (m, 3H), 1.39 (dd, J = 24.2, 12.5 Hz, 2H), 1.29 (s, 9H); ^{13}C NMR (100 MHz, DMSO-d_6) δ 160.5, 151.8, 145.6, 134.2, 133.7, 129.8, 129.1, 128.4, 127.4, 126.4, 125.6, 43.2, 42.7, 34.6, 33.8, 31.0, 26.2; MS (ESI) [M+H]$^+$ 417.3.

Scheme 2. Synthesis of compounds **2** and **3**[a]**.**

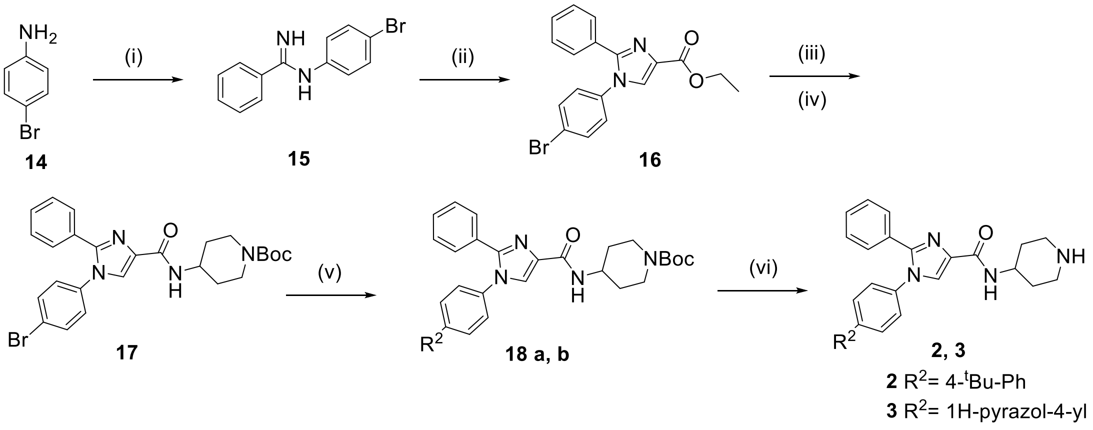

[a]*Reagents and conditions:* (i) Benzonitrile, NaH, DMSO, 0 °C to rt, 12 h, 77%; (ii) Ethyl bromopyruvate, CBr_4, MeCN, 90 °C, 12 h, 48%; (iii) NaOH, THF-H_2O(v/v, 3/1), reflux, 5 h; (iv) 4-Amino-1-Boc-piperidine, HATU, DIPEA, DMF, 5 h, 62%; (v) Arylboronic acid, $Pd(PPh_3)_4$, Na_2CO_3, *p*-dioxane-H_2O, 110 °C, 24h, 82% and 76% for **18a** and **18b**, respectively; (vi) HCl (4 N in *p*-dioxane), CH_2Cl_2, rt, 3h, 95% and 92% for **2** and **3**, respectively.

Compounds **15**, **16**, and **17** were synthesized following the same procedure as that of compounds **11-13**. Compound **17** (102 mg, 0.19 mmol), 4-tert-butylphenylboronic acid (42 mg, 0.24 mmol), tetrakis(triphenylphosphine)palladium (15 mg, 0.013 mmol), and sodium carbonate (40 mg, 0.38 mmol) in *p*-dioxane/H_2O (5/1, 6 mL) were placed in a sealed tube. The mixture was degassed and heated to 110 °C for 24 h.

The reaction was then cooled and quenched with brine (10 mL). The product was extracted with ethyl acetate (3 × 20 mL) and the combined organic layers were washed with water and brine and dried over Na_2SO_4. The volatiles were removed *in vacuo* to give a crude oil, which was purified by column chromatography (silica gel, hexanes: ethyl acetate from 2:1 to 1:1) to afford the product **18a** (90 mg, 82%) as a white foam.

To a solution of compound **18a** (90 mg, 0.16 mmol) in dichloromethane (6 mL) was added dropwise HCl (0.2 mL, 4 N in *p*-dioxane) at 0 °C. The reaction mixture was warmed to room temperature and stirred for 3 h. The volatiles were removed *in vacuo* to afford an oil, which was triturated in diether ether and solidified to give the final product **2** as a hydrochloric salt (white powder, 78 mg, 95%).

Compound 2: 1-(4'-(Tert-butyl)-[1,1'-biphenyl]-4-yl)-2-phenyl-N-(piperidin-4-yl)-1H-imidazole-4-carboxamide hydrochloride

^{1}H NMR (400 MHz, DMSO-d_6) δ 9.21 (d, *J* = 9.5 Hz, 1H), 8.98 (d, *J* = 8.5 Hz, 1H), 8.82 (d, *J* = 7.1 Hz, 1H), 8.46 (s, 1H), 7.78 (d, *J* = 8.1 Hz, 2H), 7.64 (d, *J* = 8.0 Hz, 2H), 7.52 – 7.42 (m, 7H), 7.40 (d, *J* = 7.2 Hz, 2H), 4.11 (s, 1H), 3.32 (d, *J* = 11.9 Hz, 2H), 3.01 (dd, *J* = 21.5, 11.4 Hz, 2H), 1.99 (d, *J* = 11.9 Hz, 2H), 1.89 (dd, *J* = 22.7, 11.2 Hz, 2H), 1.30 (s, 9H); ^{13}C NMR (100 MHz, DMSO-d_6) δ 159.2, 150.7, 145.7, 140.8, 135.6, 135.3, 132.4, 130.3, 129.4, 128.5, 127.5, 126.6, 126.6, 126.4, 126.1, 125.9, 44.0, 42.1, 34.3, 31.1, 28.1; MS (ESI) $[M+H]^+$ 479.3.

Compound 3: 1-(4-(1H-pyrazol-4-yl)phenyl)-2-phenyl-N-(piperidin-4-yl)-1H-imidazole-4-carboxamide hydrochloride

^{1}H NMR (400 MHz, DMSO-d_6) δ 9.12 (s, 1H), 8.94 (d, *J* = 8.6 Hz, 1H), 8.85 (s, 1H), 8.45 (s, 1H), 8.17 (s, 2H), 7.74 (d, *J* = 8.2 Hz, 2H), 7.48 – 7.35 (m, 7H), 4.16 – 4.03 (m, 1H), 3.32 (d, *J* = 11.9 Hz, 2H), 3.01 (dd, *J* = 21.1, 10.6 Hz, 2H), 1.98 (d, *J* = 11.7 Hz, 2H), 1.87 (dd, *J* = 21.3, 10.5 Hz, 2H); ^{13}C NMR (100 MHz, DMSO-d_6) δ 158.9, 145.6, 134.1, 133.7, 132.0, 131.4, 131.3, 131.3, 130.4, 129.4, 128.5, 126.5, 126.1, 125.9, 119.9, 44.0, 42.1, 28.1; MS (ESI) $[M+H]^+$ 413.2.

Scheme 3. General methods for synthesizing compounds **4, 5 and 6**[a].

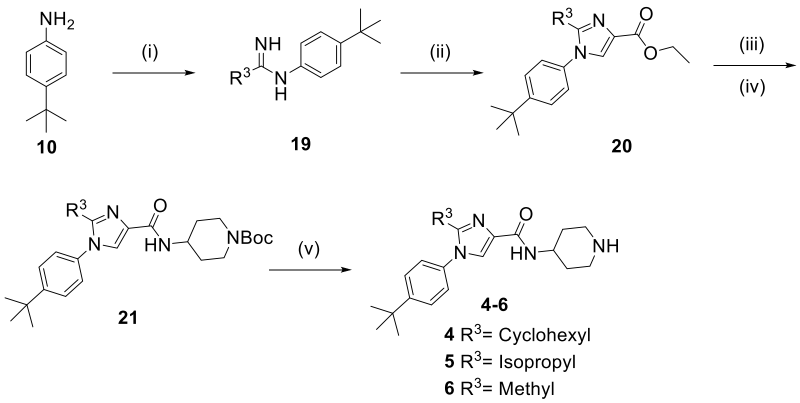

[a]*Reagents and conditions:* (i) Alkyl-nitrile, $AlCl_3$, 120 °C, 2 h, 65-81%; (ii) Ethyl bromopyruvate, CBr_4, MeCN, 90 °C, 12 h, 35-57%; (iii) NaOH, THF-H_2O(v/v, 3/1), reflux, 5 h; (iv) 4-Amino-1-Boc-piperidine, HATU, DIPEA, DMF, 5 h, 62-69% for 2 steps; (v) HCl (4 N in *p*-dioxane), CH_2Cl_2, rt, 3h, 92-95%.

To a mixture of 4-tert-butylaniline **10** (3.0 g, 20.13 mmol) and acetonitrile (1.05 mL, 20. 13 mmol) was added $AlCl_3$ (2.68 g, 20.13 mmol) in portions. The resulting slurry was stirred vigorously and heated at 120 °C for 2 h. After cooling to room temperature, the reaction was quenched with icy water, basified with sodium hydroxide (4 M solution), and extracted with dichloromethane (3 × 30 mL). The combined organic layers were washed with water and brine and dried over Na_2SO_4. The volatiles were removed *in vacuo* to give a residue, which was purified by recrystallization (hexanes/ethyl acetate) to afford **19** (3.1g, 81%) as a white solid. ^{1}H NMR (400 MHz, $CDCl_3$) δ 7.27 (d, J = 8.3 Hz, 2H), 6.76 (d, J = 7.7 Hz, 2H), 4.66 (s, 2H), 1.99 (s, 3H), 1.28 (s, 9H); ^{13}C NMR (100 MHz, $cdcl_3$) δ 155.6, 146.9, 145.3, 126.1, 121.4, 34.2, 31.6, 31.4.

Compounds **20**, **21**, **4-6** were synthesized following the same general procedure in Scheme **1**.

Compound 4: 1-(4-(Tert-butyl)phenyl)-2-cyclohexyl-N-(piperidin-4-yl)-1H-imidazole-4-carboxamide hydrochloride

^{1}H NMR (400 MHz, DMSO-d_6) δ 9.50 (s, 1H), 9.32 (s, 1H), 9.00 (d, J = 8.0 Hz, 1H), 8.62 (s, 1H), 7.67 (d, J = 8.5 Hz, 2H), 7.54 (d, J = 8.5 Hz, 2H), 4.13 – 4.08 (m, 1H), 3.33 (d, J = 11.6 Hz, 2H), 2.99 (dd, J = 21.4, 11.6 Hz, 2H), 2.65 (t, J = 11.4 Hz, 1H), 1.98 (d, J = 11.1 Hz, 2H), 1.91 – 1.79 (m, 6H), 1.72 (d, J = 11.1 Hz, 2H), 1.61 (d,

J = 8.8 Hz, 2H), 1.34 (s, 9H), 1.18 – 1.08 (m, 2H); ^{13}C NMR (100 MHz, DMSO-d_6) δ 156.5, 153.3, 152.1, 132.0, 127.3, 126.9, 125.8, 125.4, 44.1, 41.7, 34.8, 34.7, 31.0, 30.1, 27.8, 25.2, 24.7; MS (ESI) $[M+H]^+$ 409.3.

Compound 5: 1-(4-(Tert-butyl)phenyl)-2-isopropyl-N-(piperidin-4-yl)-1H-imidazole-4-carboxamide hydrochloride

^{1}H NMR (400 MHz, DMSO-d_6) δ 9.17 (s, 1H), 9.02 (s, 1H), 8.86 (s, 1H), 8.43 (s, 1H), 7.62 (d, *J* = 6.6 Hz, 2H), 7.50 (d, *J* = 6.7 Hz, 2H), 4.07 (s, 1H), 3.32 (s, 2H), 2.96 (s, 3H), 1.95 (d, *J* = 12.8 Hz, 2H), 1.87 – 1.76 (m, 2H), 1.34 – 1.20 (m, 13H); ^{13}C NMR (100 MHz, DMSO-d_6) δ 157.3, 153.7, 153.6, 132.3, 128.1, 127.2, 126.1, 124.8, 44.2, 42.1, 35.0, 31.2, 28.4, 28.0, 25.7, 20.7; MS (ESI) $[M+H]^+$ 369.3.

Compound 6: 1-(4-(Tert-butyl)phenyl)-2-methyl-N-(piperidin-4-yl)-1H-imidazole-4-carboxamide hydrochloride

^{1}H NMR (400 MHz, DMSO-d_6) δ 8.17 (s, 1H), 7.62 (d, *J* = 8.7 Hz, 2H), 7.49 (d, *J* = 8.6 Hz, 2H), 4.09 – 4.00 (m, 1H), 3.31 (d, *J* = 12.9 Hz, 2H), 2.99 (t, *J* = 11.3 Hz, 2H), 2.43 (s, 3H), 1.97 (d, *J* = 12.5 Hz, 2H), 1.78 – 1.67 (m, 2H), 1.31 (s, 9H); ^{13}C NMR (100 MHz, DMSO-d_6) δ 158.3, 153.2, 146.4, 132.9, 127.2, 125.5, 124.0, 110.0, 44.3, 42.5, 35.1, 31.4, 28.3, 12.3; MS (ESI) $[M+H]^+$ 341.2.
